# Surface proteomics and label-free quantification of *Leptospira interrogans* serovar Pomona

**DOI:** 10.1371/journal.pntd.0009983

**Published:** 2021-11-29

**Authors:** Teerasit Techawiwattanaboon, Praparat Thaibankluay, Chahya Kreangkaiwal, Suwitra Sathean-Anan-Kun, Prasong Khaenam, Jiradej Makjaroen, Trairak Pisitkun, Kanitha Patarakul

**Affiliations:** 1 Department of Microbiology, Faculty of Medicine, Chulalongkorn University, Pathumwan, Bangkok, Thailand; 2 Chula Vaccine Research Center (Chula VRC), Center of Excellence in Vaccine Research and Development, Chulalongkorn University, Pathumwan, Bangkok, Thailand; 3 Medical Science, Faculty of Medicine, Chulalongkorn University, Pathumwan, Bangkok, Thailand; 4 Center for Standardization and Product Validation, Faculty of Medical Technology, Mahidol University, Bangkok-Noi, Bangkok, Thailand; 5 Center of Excellence in Systems Biology, Research Affairs, Faculty of Medicine, Chulalongkorn University, Pathumwan, Bangkok, Thailand; Baylor College of Medicine, UNITED STATES

## Abstract

Leptospirosis is a re-emerging zoonosis with a global distribution. Surface-exposed outer membrane proteins (SE-OMPs) are crucial for bacterial–host interactions. SE-OMPs locate and expose their epitope on cell surface where is easily accessed by host molecules. This study aimed to screen for surface-exposed proteins and their abundance profile of pathogenic *Leptospira interrogans* serovar Pomona. Two complementary approaches, surface biotinylation and surface proteolytic shaving, followed by liquid chromatography tandem-mass spectrometry *(*LC-MS/MS) were employed to identify SE-OMPs of intact leptospires. For quantitative comparison, in-depth label-free analysis of SE-OMPs obtained from each method was performed using MaxQuant. The total number of proteins identified was 1,001 and 238 for surface biotinylation and proteinase K shaving, respectively. Among these, 39 were previously known SE-OMPs and 68 were predicted to be localized on the leptospiral surface. Based on MaxQuant analysis for relative quantification, six known SE-OMPs including EF- Tu, LipL21, LipL41, LipL46, Loa22, and OmpL36, and one predicted SE-OMPs, LipL71 were found in the 20 most abundant proteins, in which LipL41 was the highest abundant SE-OMP. Moreover, uncharacterized LIC14011 protein (LIP3228 ortholog in serovar Pomona) was identified as a novel predicted surface βb-OMP. High-abundance leptospiral SE-OMPs identified in this study may play roles in virulence and infection and are potential targets for development of vaccine or diagnostic tests for leptospirosis.

## Author summary

Surface-exposed outer membrane proteins (SE-OMPs) are important components of pathogenic *Leptospira* mediating virulence and interacting with host cells and host environment. This study aimed to identify SE-OMPs of pathogenic *L*. *interrogans* serovar Pomona using surface biotinylation and proteinase K (proK) shaving methods comprehensively. Surface-protein enriched fractions obtained from intact leptospires were subsequently subjected to liquid chromatography tandem-mass spectrometry (LC-MS/MS) and their label-free quantitative profiles were analyzed by MaxQuant software. The surface biotinylation and proK shaving yielded a total of 1,001 and 238 proteins, respectively, and shared 220 proteins. Of these, 28 known SE-OMPs were identified by either method and 11 known SE-OMPs were overlapped. Moreover, 50 predicted SE-OMPs were detected in individual sample group and 18 were common in both groups. In the 20 most abundant proteins, there were six known SE-OMPs derived from both methods, including EF- Tu, LipL21, LipL41, LipL46, Loa22, and OmpL36, and one predicted SE-OMP, LipL71. McpA, OmpL1, OmpL32, OmpL36, SdhA, SppA, LIC10314, and LIC12615 were ranked in the 50 most abundant proteins. Furthermore, LIC10411 (LIP3228 ortholog in serovar Pomona) was predicted as a novel surface βb-OMP. The high-abundance SE-OMPs should be further investigated as novel vaccine candidates or diagnostic biomarkers for leptospirosis.

## Introduction

Leptospirosis is a neglected zoonosis with high global prevalence, mainly in tropical and subtropical regions including Southeast Asia, Oceania, the Indian subcontinent, Caribbean, and Latin America [1,2]. Leptospirosis causes approximately 1.03 million cases and 58,900 deaths annually worldwide [3]. The severity of the disease ranges from asymptomatic infection to severe manifestations with multi-organ dysfunction, such as renal and hepatic failure, pulmonary hemorrhage, and myocarditis [2]. The major etiologic agent of the illness is pathogenic *Leptospira* spp., but some cases are associated with intermediate species such as *L*. *inadai* and *L*. *wolffii* [4]. Most wild and domestic animals can be reservoir hosts harboring the pathogens in their kidneys. Humans are considered as accidental hosts by direct contact with leptospires shed in urine of infected animals or indirect exposure to water or soil contaminated with the urine [2].

Bacterial outer membrane proteins (OMPs) often play key roles in bacterial pathogenesis such as adhesins, porins, targets for antibodies, and receptors for various host molecules. OMPs are likely to be crucial for adaptation and response to host conditions and surrounding environments [5]. Surface-exposed (SE) domains of OMPs on pathogenic leptospires are important because of their location on a tip of cell surface where facilitates bacterial-host interactions [6]. Identification of leptospiral SE-OMPs should be helpful not only for a better understanding of *Leptospira* pathogenicity but also new targets for the development of vaccines and diagnostic tests [7]. A variety of surface protein assessment methods have been established for isolation of bacterial surface-associated proteins [8,9]. Surface biotinylation method using hydrophilic biotins with membrane impermeable properties can label surface proteins on intact cells [10–12]. As a complementary approach, proteolytic shaving of surface proteins under conditions that preserve cell integrity can harvest SE-OMPs [12–14]. Proteinase (proK) has broad *cleavage sites resulting in coverage of most surface proteins [9].* Pinne and Haake previously used surface biotinylation and surface shaving with proK to identify new 4 surface-exposed leptospiral OMPs [12]. The quantitative profile on peptides or proteins from different biological samples and conditions become important for advanced medical research. MaxQuant is one of the most widely used computational software to quantify protein from mass spectral peak intensity to reveal protein abundance profiling [15]. This software is easy-to-use, and available for label-free quantification (LFQ) and analysis of data obtained from most MS platforms.

In this study, a combination of surface biotinylation and surface shaving methods followed by liquid chromatography tandem-mass spectrometry (LC-MS/MS) were used for high-throughput identification of SE-OMPs on *L*. *interrogans* serovar Pomona. The results were subsequently analyzed by MaxQuant software for quantitative profiling of leptospiral SE-OMPs. Our findings revealed known and putative SE-OMPs and the abundance profile of pathogenic *Leptospira*.

## Materials and methods

### *Leptospira* and culture

*Leptospira interrogans* serovar Pomona, kindly provided by Ben Adler, was originally from Lee Smythe, World Health Organization/Food and Agricultural Organization/Office International des Epizooties Collaborating Centre for Reference and Research on Leptospirosis, Queensland Health Scientific Services, Australia. All experiments used low-passage leptospires, which was directly isolated from infected hamsters [16] followed by less than five *in vitro* passages. *Leptospira* was cultivated at 30°C in Ellinghausen-McCullough-Johnson-Harris (EMJH) medium (Difco, MD, USA) containing 10% bovine serum albumin (BSA) supplement solution [17] until the log phase (~5×10^8^ cells/ml) was reached.

### Preparation of leptospiral surface proteins

The surface biotinylation and proteinase K shaving assays were performed following previously described protocols with some modifications [12]. Leptospires were harvested by low-speed centrifugation at 2,000×g for 7 min at room temperature (RT). The leptospiral cell pellets were gently washed twice with BSA-free EMJH base medium and approximately 1×10^10^ cells were prepared for each method as follows.

#### Cell surface biotinylation

The surface biotinylation was carried out using Pierce Cell Surface Protein Biotinylation and Isolation Kit (Thermo Scientific) according to the manufacturer’s instruction. The cell pellets were resuspended with BupH Phosphate Buffered Saline (BupH-PBS) containing Sulfo-NHS-SS-Biotin at various concentrations up to 1 mg/ml. After incubation at RT for 30 min, the reaction was stopped with BupH-PBS containing 100mM glycine and the inactivated biotin was removed by washing twice with BupH-PBS. The labeled pellets were resuspended with BupH-PBS containing cOmplete protease inhibitors cocktail (Roche) before sonication on ice for 30 min using Ultrasonic Processor (GE Healthcare, Buckinghamshire, UK) (pulse on for 15 sec, pulse off for 45 sec, 35% amplitude). The cell lysates were centrifuged at 15,000×g for 5 min at 4°C and biotin-labeled proteins in the supernatant were purified using NeutrAvidin Agarose column according to the manufacturer’s instruction. The eluted proteins were collected for LC-MS/MS.

#### Cell surface shaving with proteinase K

The cell pellets were resuspended with Proteinase K Solution (Promega, Madison, WI) at various concentrations up to 2 μg/ml. After incubation at 37°C for 30 min, the reactions were stopped with cOmplete protease inhibitors cocktail (Roche) solution. The cell lysates were centrifuged at 15,000×g at 4°C for 5 min and the supernatant containing cleaved surface proteins was collected for LC-MS/MS analysis. The remaining cell pellets were subjected to SDS-PAGE and Western blotting.

### Live/Dead fluorescence viability staining

Cell membrane integrity was determined using LIVE/DEAD BacLight Bacterial Viability Kit (Thermo Scientific) according to the manufacturer’s instruction. Equal volumes of SYTO9 and propidium iodide (PI) were mixed. The biotinylated cells and the remaining cell pellets after proK shaving were incubated with dye mixture at a ratio of 1:1,000 in the dark at RT for 15 min. The stained cells were observed under a fluorescence microscope (Olympus) at 400× magnification. Leptospiral cells treated with cold absolute methanol for 5 min on ice were used as control cells with compromised membrane.

### Sodium dodecyl sulphate–polyacrylamide gel electrophoresis

Protein samples were prepared following previously described protocols [18] except biotin-labeled samples were mixed with 1× sample buffer without reducing agent. The samples were characterized by 15% polyacrylamide gel and stained with Coomassie Brilliant Blue R-250 (Bio-Rad, Germany).

### Western blotting

Proteins on polyacrylamide gel were transferred onto nitrocellulose membrane. After incubating with 1% BSA in PBS plus 0.05% Tween-20 (PBST) at RT for 1 h, the membrane was incubated with polyclonal antibodies (kindly provided by David A. Haake, UCLA) against OmpL1 (1: 2,000) and FlaA1 (1: 2,000) at RT for 1 h. Afterwards, the membrane was incubated with horseradish peroxidase (HRP)-conjugated goat anti-rabbit IgG (1: 5,000, KPL, MD, USA) at RT for 1 h. For biotinylated proteins, the membrane was incubated with HRP -conjugated streptavidin (1:5000; BD Pharmingen). After each incubation, the membrane was washed three times for 5 min with PBST. The protein bands were detected with ECL chemiluminescent substrate (Amersham ECL Prime, GE Healthcare) under ChemiDoc XRS+ System (Bio-Rad).

### In-gel digestion

Protein samples were prepared for mass spectrometry using previously described protocols [18,19] with some modifications. The entire sample lane on Coomassie Brilliant Blue R-250 stained polyacrylamide gel was diced into approximately 1 mm^3^ pieces and destained three times for 10 min with 25 mM ammonium bicarbonate (Ambic) in 50% acetonitrile (ACN). The gel pieces were dehydrated with 100% ACN for 5 min and completely dried by a speed vacuum device (Thermo scientific). The samples were reduced with 10 mM DTT in 25 mM Ambic at 56°C for 45 min and alkylated with 55 mM iodoacetamide in 25 mM Ambic at RT for 30 min in the dark. The gel pieces were dehydrated and completely dried before incubating with Sequencing Grade Modified Trypsin (Promega) on ice for 60 min. The excess solution was replaced with 25 mM Ambic and the protein samples were incubated at 37°C overnight. The digested peptides were extracted from the gel pieces with 50% ACN in 0.1% formic acid (FA) and dried *in vacuo*. The dried samples were acidified with 0.1% FA and desalted with C-18 spin columns (Thermo Scientific) according to the manufacturer’s instruction. The dried peptides were finally resuspended in 0.1% FA before applying to LC-MS/MS.

### Liquid chromatography tandem-mass spectrometry

Peptide analysis by LC-MS/MS was performed using an EASY-nLC1000 system (Thermo Scientific) coupled to a Q-Exactive Orbitrap Plus mass spectrometer (Thermo Scientific) equipped with a nano-electrospray ion source (Thermo Scientific). The peptides were eluted in 5–20% acetonitrile in 0.1% FA for 40 min followed by 20–40% acetonitrile in 0.1% FA for 10 min and 40–98% acetonitrile in 0.1% FA for 10 min at a flow rate of 300 nl/min. The MS methods included a full MS scan at a resolution of 70,000 followed by 10 data-dependent MS2 scans at a resolution of 17,500. The normalized collision energy of HCD fragmentation was set at 32%. An MS scan range of 350 to 1400 m/z was selected and precursor ions with unassigned charge states, a charge state of +1, or a charge state of greater than +8 were excluded. A dynamic exclusion of 30 s was used.

### Protein identification and label-free quantification by MaxQuant

Protein identification and label-free quantitative analysis were performed using MaxQuant software suite version 1.6.1.0 and its built-in Andromeda search engine. The mass spectra data (raw file format) derived from LC-MS/MS was searched against the whole *L*. *interrogans* serovar Copenhageni Fiocruz L1-130 protein database available in Uniprot database (www.uniprot.org, taxonomy = 267671). The data (three independent experiments) from each surface protein assessment method were analyzed in separate MaxQuant run for protein identification. In addition, three biological replicates from each method were assigned to different experiments and analyzed in the same software run for quantitative proteomics. The default parameters for LFQ were used with some additional settings as follows: Group specific parameters; Instrument = Orbitrap; Digestion = Specific with Trypsin/P; Label free quantification = LFQ with LFQ min. ratio count = 2, Fast LFQ was selected, LFQ min. number of neighbors = 3, LFQ average number of neighbors = 6. The MaxQuant output file (proteinGroups.txt format) was further analyzed using Microsoft Excel. Any contaminants and reverse identified proteins were removed from the total proteins list. The proteins repeatedly identified by at least 2 out of 3 replicates were considered proteins that were truly yielded by each method. The results of known leptospiral SE-OMPs retrieved from PubMed and Scopus are shown in S1 Table.

### Bioinformatics tools

The amino acid sequences of LIP3483, LIP3482, LIP3334, LIP3228, LIP3144, LIP1773, LIP2297, LIP0991, and LIP3761 proteins in *L interrogans* serovar Pomona were retrieved from the Victorian Bioinformatics Consortium (https://vicbioinformatics.com/) in fasta file format. Subcellular localization was predicted using PSORTb v3.0.3 [20], CELLO v.2.5 [21], GNeg-mPLoc v. 2.0 [22], and SOSUI-GramN [23]. Lipoprotein was predicted using LipoP v. 1.0 [24]. The presence of signal peptides was predicted using SignalP v. 4.0 [25], Signal-CF [26], and PrediSi [27]. Transmembrane α helix was predicted using TMHMM v. 2.0 [28], Phobius [29], and CCTOP [30]. The β-barrel (βb) OMPs were predicted using HHomp [31], PRED-TMMB [32], and TMBETADISC-RBF [33]. All predictions were performed with default settings of Gram-negative bacteria and the criteria for identifying of SE-OMPs followed the previously described criteria [34]. βb-OMP was defined as a protein containing a signal peptide, a transmembrane α-helix lower than 2, and a βb transmembrane domain. OM lipoprotein was defined based on predicted location in the OM and containing a lipoprotein signal peptide.

## Results

### Optimization of cell surface biotinylation with Sulfo-NHS-SS-Biotin

**Fig 1:**
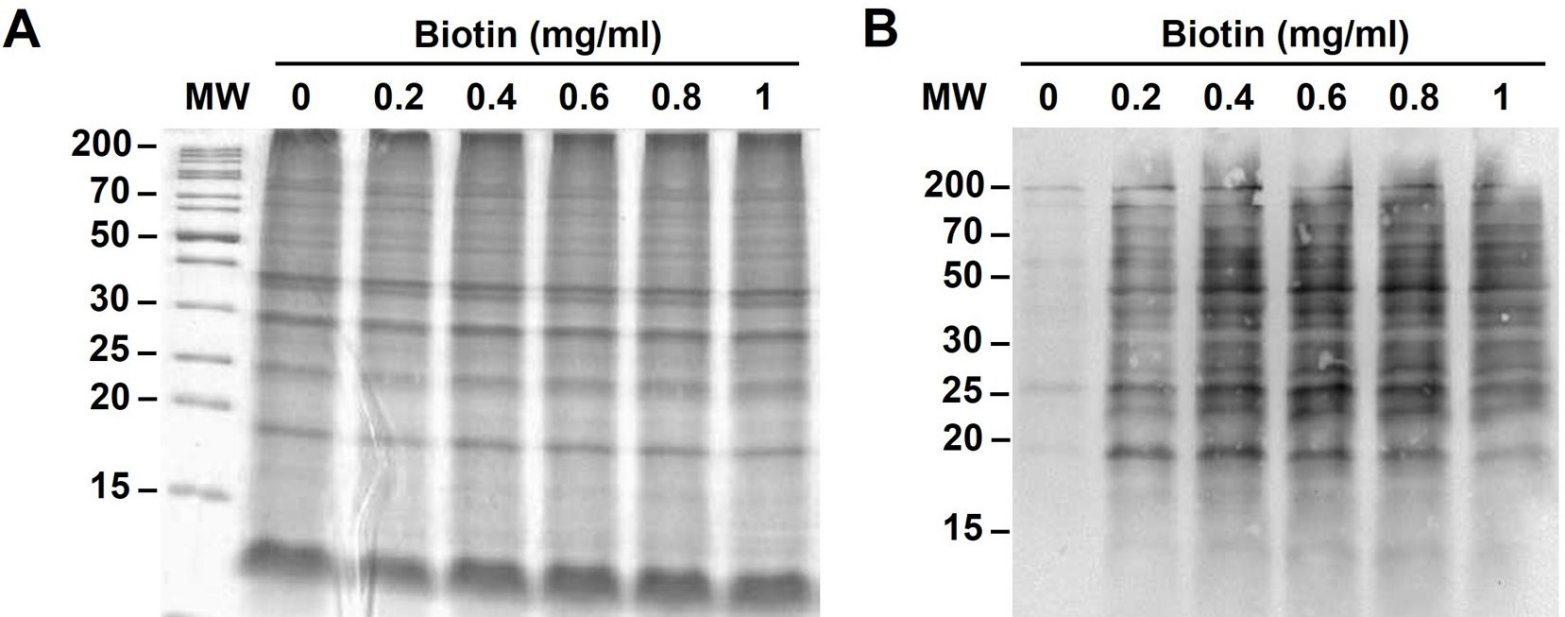
Optimization of surface biotinylation of intact *Leptospira*. Intact leptospires (~1×10^10^ cells) were incubated with Sulfo-NHS-SS-Biotin at a final concentration of 0, 0.2, 0.4, 0.6, 0.8 and 1 mg/ml. The biotinylated proteins from approximately 10^8^ cells were loaded per lane, separated by SDS-PAGE, and stained with Coomassie Brilliant Blue R-250 (A). The biotinylated proteins were transferred to a nitrocellulose membrane, stained with 1:5,000 HRP-conjugated streptavidin, and detected with ECL chemiluminescence detection system (B). The intact leptospires in PBS (0 mg/ml biotin) were used as a negative control. The position of PageRuler Unstained Protein Ladder (Thermo Scientific) are indicated to the left (lane MW).

**Fig 2:**
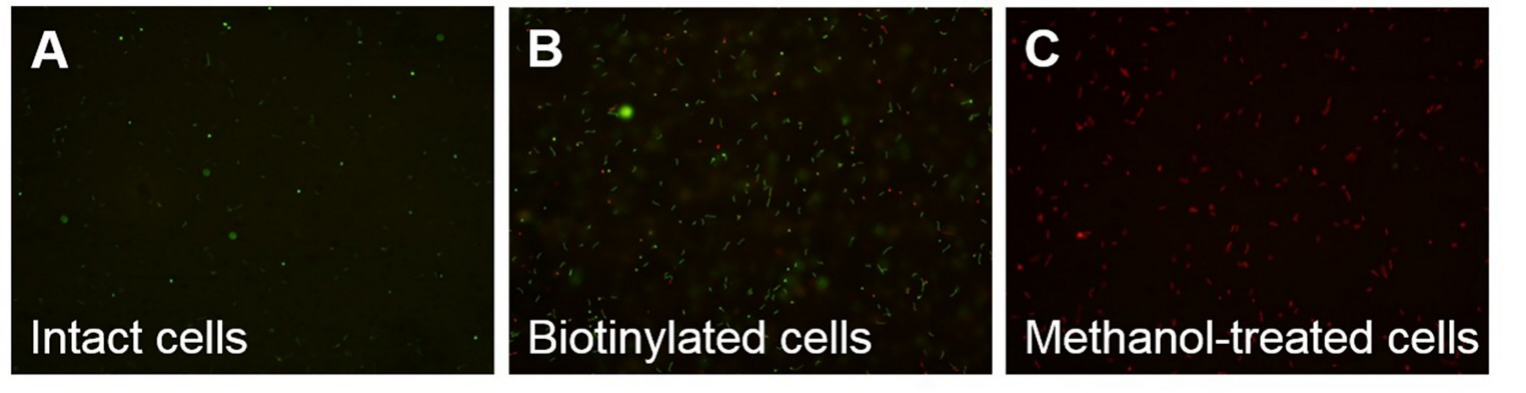
Determination of cell membrane integrity after surface biotinylation. Membrane integrity of intact leptospires was determined by Live/Dead (SYTO9/PI) fluorescence viability staining. The intact cells before (A) and after (B) labeling with 0.4 mg/ml Sulfo-NHS-SS-Biotin, and methanol-treated cells used as non-intact cell control (C) were stained with Live/Dead (SYTO9/PI) fluorescent dyes and visualized under a fluorescence microscope. The green (SYTO9) and red (PI) colors indicate intact cells and membrane disrupted cells, respectively.

Initially, the concentration of Sulfo-NHS-SS-Biotin was optimized for surface protein labeling. SDS-PAGE showed approximately equal amounts of total protein used for each concentration of biotin (Fig 1A). Band intensities of biotinylated proteins on the Western blot reached the highest signal when the biotin was used at the final concentration of 0.4 mg/ml (Figs 1B and S1), therefore subsequent labeling was performed using 0.4 mg/ml biotin. Only minimal signals were seen in the unlabeled control (0 mg/ml of biotin). The labeling process might disrupt the surface layer and potentially release proteins from interior compartments. The Live/Dead fluorescence viability staining was performed to determine membrane integrity before and after surface biotinylation. Most leptospires were stained with SYTO9 (green), which indicated intact cells, before (Fig 2A) and after (Fig 2B) biotin labeling suggesting that the membrane integrity was mainly preserved after the labeling process. In contrast, all leptospires treated with methanol used as control cells with damaged membrane were stained red with PI as expected (Fig 2C).

**Fig 3:**
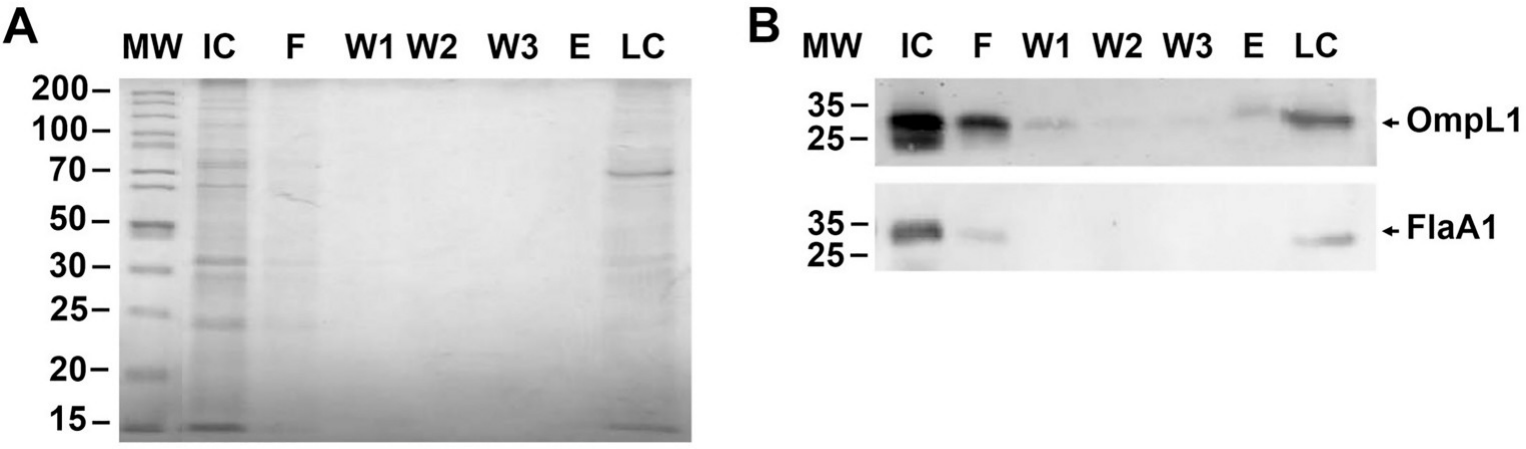
Purity of surface proteins after biotinylation and purification. Intact leptospires (~1×10^10^ cells) were incubated with Sulfo-NHS-SS-Biotin at a final concentration of 0.4 mg/ml. The biotinylated proteins from intact *Leptospira* cells (IC) were loaded onto an avidin agarose column for purification. Non-biotinylated proteins were discarded to flow-through fraction (F). The column was washed 3 times (W1-W3) and the purified proteins were finally eluted (E). Each fraction at equal volume was separated by SDS-PAGE and stained with Coomassie Brilliant Blue R-250 (A) or transferred to nitrocellulose membranes and detected with polyclonal rabbit antisera against OmpL1 (~31kDa) and FlaA1 (~35kDa), known SE-OMP and periplasmic proteins, respectively (B). The biotinylated lysate cells (LC) were used as a positive control. The expected sizes of OmpL1 and FlaA1 are indicated on the right, and the position of PageRuler Unstained Protein Ladder (Thermo Scientific) is indicated on the left (lane MW).

After surface labeling and purification through the avidin agarose column, the purity of surface proteins in the eluted fraction was determined by immunoblotting using antisera against OmpL1 and FlaA1, known leptospiral SE-OMP and periplasmic proteins, respectively (Fig 3). OmpL1 but not FlaA1 was detected in the eluted fraction (S2 Fig), indicating that biotinylation optimally occurred on the cell surface, and the purified proteins mainly contained OMPs. The surface-exposed proteins were further analyzed by LC-MS/MS.

### Optimization of cell surface shaving with proteinase K treatment

**Fig 4:**
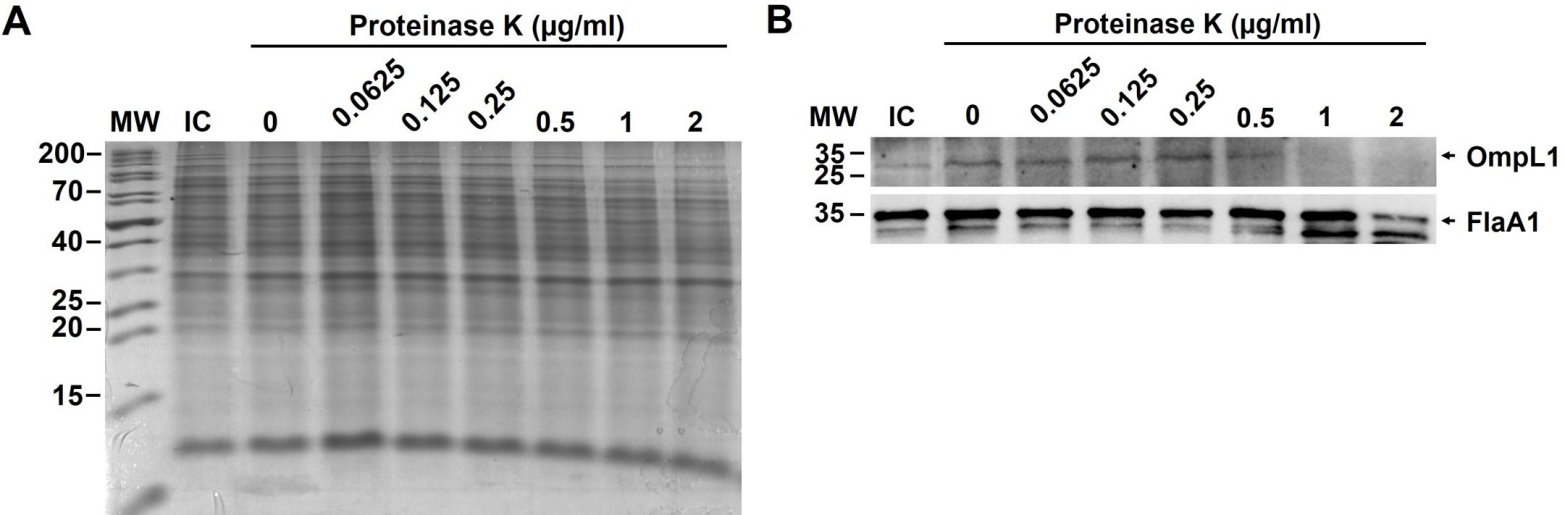
Optimization of leptospiral cell surface shaving with proteinase K. Approximately 1×10^10^ cells of intact leptospires (IC) were incubated with proK at a final concentration of 0, 0.0625, 0.125, 0.25, 0.5, 1, and 2 μg/ml. Equivalents of proK-treated 10^8^ leptospires per lane were separated by SDS-PAGE and Coomassie Brilliant Blue staining (A), or transferred to a nitrocellulose membrane and probed with polyclonal rabbit antisera against OmpL1 (~31kDa) and FlaA1 (~35kDa), known SE-OMP and periplasmic protein, respectively. The expected sizes of OmpL1 and FlaA1 are indicated on the right and the position of PageRuler Unstained Protein Ladder (Thermo Scientific) is indicated on the left (lane MW).

**Fig 5:**
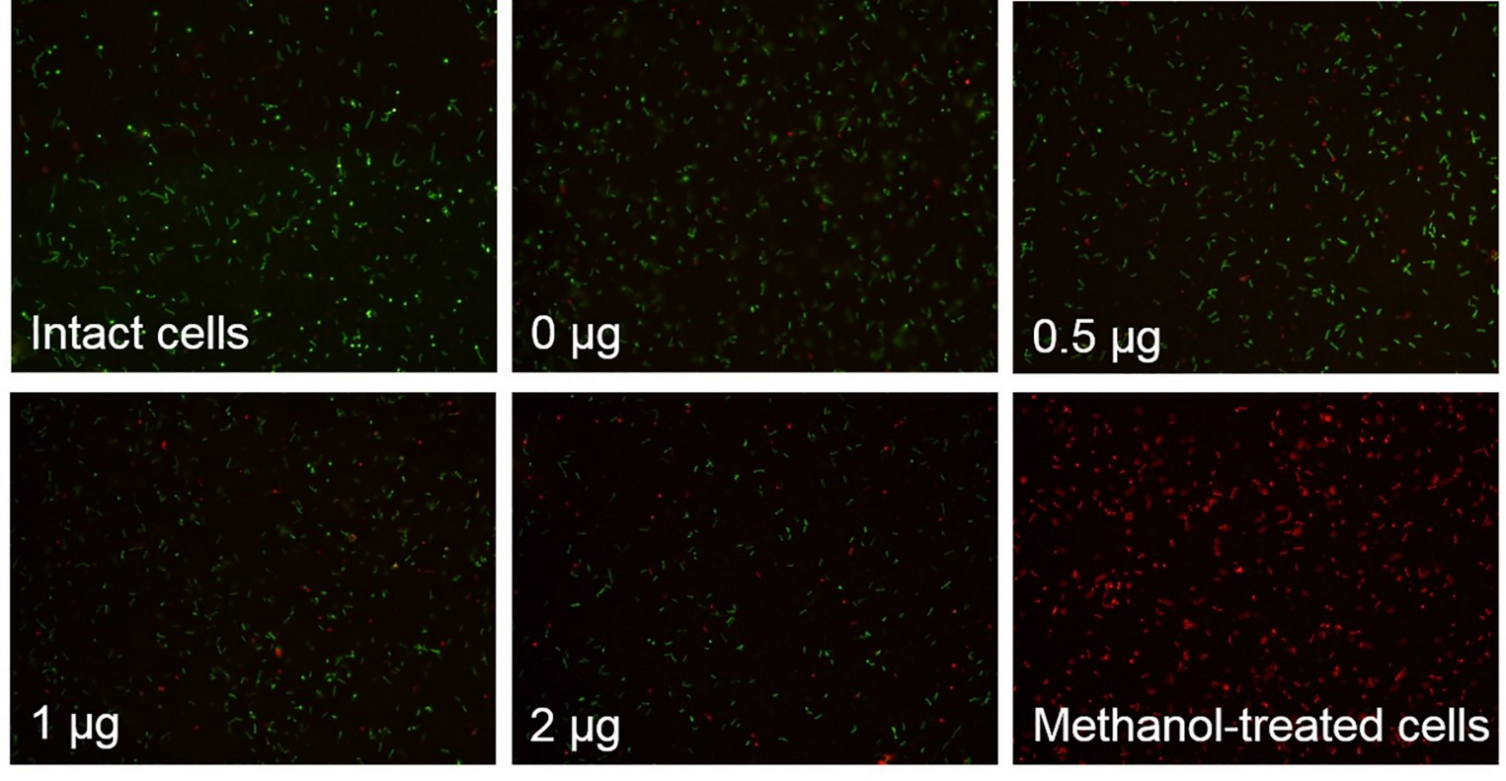
Determination of membrane integrity after cell surface shaving. Membrane integrity of intact leptospires was determined by Live/Dead (SYTO9/PI) fluorescence staining. The intact cells before and after treatment with proK at various concentrations of 0, 0.0625, 0.125, 0.25, 0.5, 1, and 2 μg/ml, and methanol-treated cells used as non-intact cell control were stained with Live/Dead (SYTO9/PI) fluorescent dyes and visualized under a fluorescence microscope. The green (SYTO9) and red (PI) colors indicate intact cells and membrane disrupted cells, respectively.

The optimal concentration of proK that cleaved only surface proteins and still maintained cell membrane integrity was determined. After proK treatment, the presence of OmpL1 and FlaA1 in the cell pellets containing shaved cells was determined by immunoblotting. When a comparable amount of each sample was used (Fig 4A), the band intensity of OmpL1 but not FlaA1 was reduced at 1 μg/ml proK treatment (Fig 4B) indicating that surface proteins were cleaved with the least effect on periplasmic proteins. The cell viability staining with SYTO9/PI showed that lytic cells increased as a dose-dependent manner (Fig 5). Most leptospiral cells remained intact after treatment with 1 μg/ml proK. Therefore, proK treatment at a final concentration of 1 μg/ml was used to cleave SE-OMPs from remaining intact cells. The supernatant portion obtained from treated cells was used to identify SE-OMPs by LC-MS/MS.

### Identification of proteins from surface biotinylation and proteinase K shaving by LC-MS/MS

**Fig 6:**
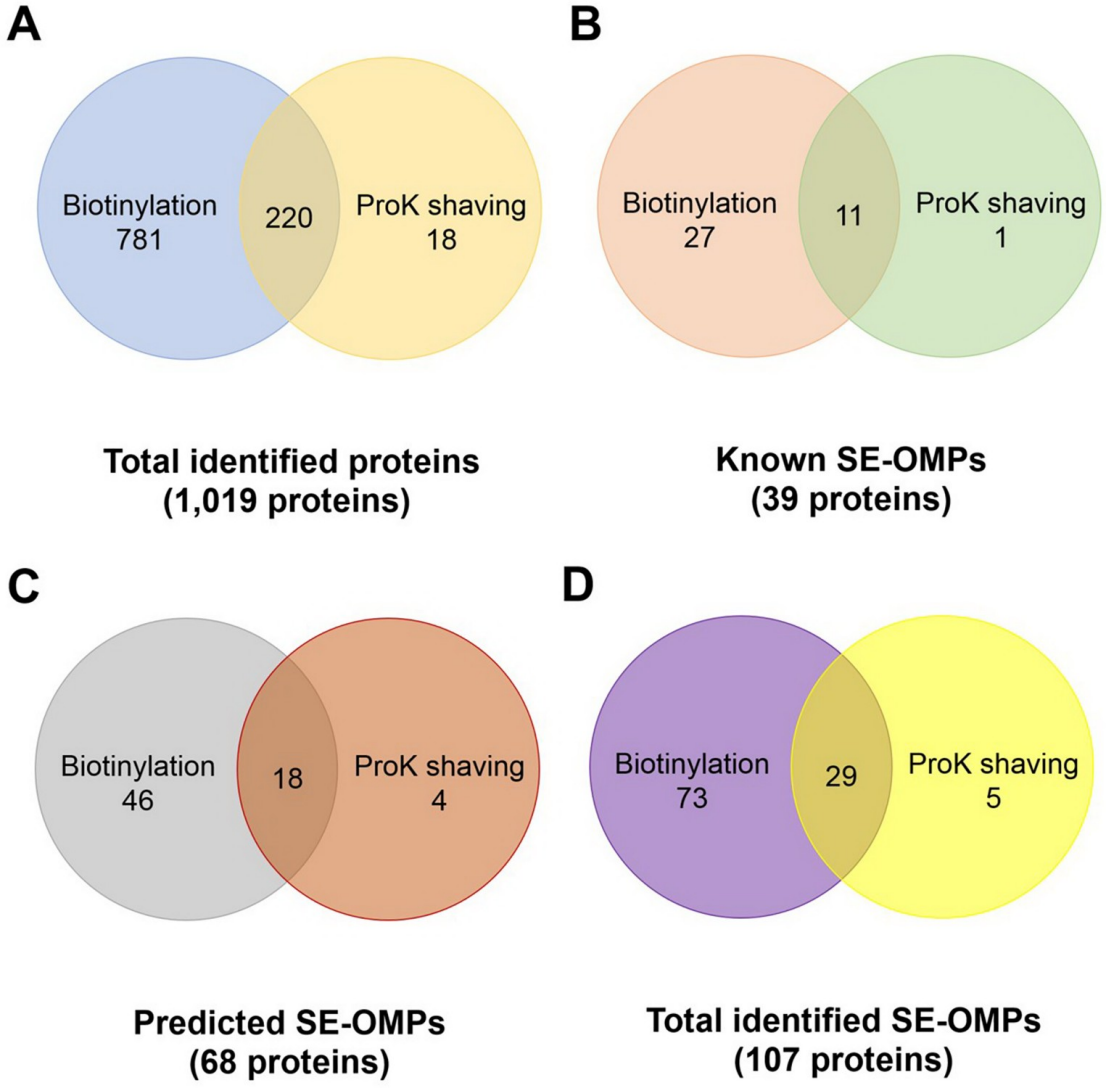
The number of identified proteins obtained by surface biotinylation and surface shaving. The number of total identified proteins (A), known SE-OMPs (B), predicted SE-OMPs (C), and total SE-OMPs obtained by surface biotinylation and surface shaving. The protein samples were prepared by surface biotinylation and proK shaving before identification by LC-MS/MS. The mass spectrum data were analyzed by MaxQuant software and its built-in Andromeda search engine. The data were searched against the whole protein database of *Leptospira interrogans* serovar Copenhageni Fiocruz L1-130. Any contaminants and reverse identified proteins were removed from the total identified proteins list. The relevant articles in leptospiral SE-OMPs were retrieved from PubMed and Scopus (S1 and S2 Tables).

Surface biotinylation and proK shaving of intact *L*. *interrogans* serovar Pomona were subsequently performed according to the optimized protocols. Due to unavailable protein database of serovar Pomona, the mass spectrum data were searched against the Uniprot database of *L*. *interrogans* serovar Copenhageni Fiocruz L1-130 containing a total of 3,655 proteins. After MaxQuant analysis, the biological replicates were normalized and averaged the data (mean of two or three data) to obtain a single data set of LFQ intensity from each method (S1 Appendix). The analysis demonstrated that surface biotinylation yielded a total of 1,001 proteins, proK shaving yielded a total of 238 proteins, and both methods identified a total of 1,019 proteins (Fig 6A). The biotinylation and proK shaving identified unique 781 and 18 proteins, respectively, while 220 proteins overlapped between the two methods. Previously reported 39 SE-OMPs were identified by at least one method (S1 Table). The number of known SE-OMPs identified by biotinylation and proK surface shaving were 38 and 12, respectively. Of these, 11 known SE-OMPs were shared by both sample groups (Fig 6B).

According to the previous studies on reverse vaccinology, two bioinformatics approaches predicted a total of 272 leptospiral SE-OMPs [34,35], of which 23 proteins were known SE-OMPs but 2 proteins were currently missing from the database. Of 247 remaining proteins, there were 68 predicted SE-OMPs identified by at least one method (S2 Table). The predicted 64 and 22 SE-OMPs were detected in biotinylated and proK-shaved samples, respectively (Fig 6C). The number of predicted SE-OMPs overlapped in both sample groups was 18. Based on this information, total known and predicted SE-OMPs obtained from both techniques accounted for 10.50% (107/1,019) of all identified proteins. Of these, 29 known and predicted SE-OMPs represented 13.19% (29/220) of total identified proteins shared by both methods (Fig 6D).

### Quantitative surface proteomics of pathogenic *Leptospira*

The quantitative profiling of protein abundance was presented as the abundance ranking of LFQ intensity of all identified proteins obtained from each method. The quantitative abundance profiling of known SE-OMPs and predicted SE-OMPs are shown in S1 and S2 Tables, respectively, while the 20 most abundant proteins of each method are shown in Tables 1 and 2. The highest abundant SE-OMP was LipL41, whatever the method used. Known SE-OMPs including EF-Tu, LipL21, LipL46, Loa22, and OmpL36, and predicted SE-OMP, LipL71 were shown in the top 20 high abundance, in which EF-Tu, LipL46, and OmpL36 were overlapping proteins. Moreover, there were 9 known SE-OMPs and 5 predicted SE-OMPs ranked in the top 50 high abundance (S3 Table). Of these, 8 proteins including EF-Tu, LipL21, LipL41, LipL46, LipL71, McpA, OmpL1, and OmpL36 were common in both sample groups while Loa22, OmpL32, SdhA, SppA, LIC10314, and LIC12615 were specific in each group. However, outer membrane proteins (LipL31, LipL32, and LipL45), periplasmic proteins such as flagellin (FlaA-1 and FlaA-2), cytoplasmic proteins such as global regulator (Rpo family and elongation factor), metabolic enzymes, chaperone proteins (GroEL and DnaK), and proteins with unknown functions were also presented in high quantity.

**Table 1:** The 20 most abundant proteins identified by surface biotinylation.

Abundance ranking^a^	Gene ID	Gene names	Protein IDs	Protein names
1	LIC11335	*groEL*	P61438	GroEL
2	LIC11352	*lipL32*	Q72SM7	LipL32
**3**	**LIC12966**	***lipL41***	**Q72N71**	**LipL41**
4	LIC12407	*glnA*	Q72PR0	Putative glutamine synthetase protein
5	LIC13432	*lic13432*	Q72LW1	Uncharacterized protein
6	LIC10403	*ribH*	P61724	6,7-dimethyl-8-ribityllumazine synthase
**7**	**LIC10191**	***loa22***	**Q72VV5**	**Loa22**
**8**	**LIC13166**	***ompL36***	**Q72MM7**	**OmpL36**
9	LIC12233	*lic12233*	Q72Q79	Fructose-bisphosphate aldolase
10	LIC11687	*lic11687*	Q72RQ7	Endonuclease
11	LIC11652	*tal*	Q72RT8	Probable transaldolase
12	LIC11890	*lic11890*	Q72R58	Flagellin
13	LIC11456	*lipL31*	Q72SC8	LipL31
**14**	**LIC12875**	***tuf***	**Q72NF9**	**Elongation factor Tu (EF-Tu)**
**15**	**LIC11885**	***lipL46***	**Q72R63**	**LipL46**
16	LIC11194	*lic11194*	Q72T27	Putative citrate lyase
17	LIC10483	*lic10483*	Q72V20	Uncharacterized protein
18	LIC10874	*lic10874*	Q72TZ0	Molybdopterin oxidoreductase
19	LIC11243	*atpD*	Q72SX9	ATP synthase subunit beta
20	LIC11643	*lic11643*	Q72RU5	LipL45

^a^ The ranking was calculated throughout all identified proteins of surface biotinylation.

Bold fonts imply overlapping proteins.

**Table 2:** The 20 most abundant proteins identified by proteinase K shaving.

Abundance ranking^a^	Gene ID	Gene names	Protein IDs	Protein names
1	LIC13328	*lic13328*	Q72M63	Isocitrate dehydrogenase
2	LIC11335	*groEL*	P61438	GroEL protein
3	LIC12082	*cysK*	Q72QN1	Cysteine synthase
**4**	**LIC12966**	***lipL41***	**Q72N71**	**LipL41**
5	LIC11352	*lipL32*	Q72SM7	LipL32
6	LIC10175	*lic10175*	Q72VX0	Uncharacterized protein
**7**	**LIC11003**	***lipL71***	**Q72TL5**	**LipL71**
8	LIC11517	*accA2*	Q72S69	Acetyl-CoA carboxylase alpha subunit
9	LIC11359	*maoC*	Q72SM0	MaoC
10	LIC10754	*rpoC*	Q72UA7	DNA-directed RNA polymerase subunit beta
**11**	**LIC12875**	***tuf***	**Q72NF9**	**Elongation factor Tu (EF-Tu)**
12	LIC11781	*mdh*	P61975	Malate dehydrogenase
**13**	**LIC10011**	***lipL21***	**Q72WC6**	**LipL21**
14	LIC13470	*lic13470*	Q72LT0	Ferredoxin—NADP reductase
15	LIC10753	*rpoB*	Q72UA8	DNA-directed RNA polymerase subunit beta
16	LIC11602	*lic11602*	Q72RY6	GSDH domain-containing protein
17	LIC10524	*dnaK*	P61442	Chaperone protein DnaK
**18**	**LIC13166**	***ompL36***	**Q72MM7**	**OmpL36**
**19**	**LIC11885**	***lipL46***	**Q72R63**	**LipL46**
20	LIC10002	*dnaN*	Q72WD5	Beta sliding clamp

^a^ The ranking was calculated throughout all identified proteins of surface proK shaving.

Bold fonts imply overlapping proteins.

### Subcellular localization prediction of high-abundance unknown proteins

The uncharacterized proteins; LIC10175, LIC10176, LIC10314, LIC10411, LIC10483, LIC11182, LIC11848, LIC12621, LIC13432, were ranked in the top 50 high abundance proteins (S3 Table). They were further predicted to be SE-OMPs using web-based bioinformatics tools following previously described criteria [34]. The amino acid sequences of those uncharacterized proteins in serovar Pomona were used for predictions. The prediction demonstrated that LIP3228 (an orthologous protein of LIC10411) contained a signal peptide and a βb structure but lacked a transmembrane α-helix (S4 Table). Therefore, LIP3228 was finally predicted to be a βb-OMP. However, the remaining 8 uncharacterized proteins were not predicted as SE-OMPs based on those criteria.

## Discussion

The location on the exterior of cell surface enables SE-OMPs of pathogens to promptly interact with host molecules and surrounding environments. SE-OMPs of pathogenic leptospires play roles in virulence, including adherence, invasion, colonization, and interaction with the host environment, including the immune system [6].

Several methods were previously used to identify leptospiral SE-OMPs, such as cell surface labeling [10–12], surface proteolysis shaving [12–14], surface immunofluorescence [12,36], surface immunoprecipitation [37], and *in silico* analysis [12,34,35]. Of these, surface labeling with biotin and surface shaving with proteinase K can be used as high-throughput screening to identify SE-OMPs. Both methods are practical and reliable for identification of surface proteins of leptospires [10–14] and other bacterial pathogens [38–41]. In this study, we used two complementary methods, surface biotinylation, and proK shaving previously used to characterize novel leptospiral SE-OMPs [12], to identify proteins localized on the surface of *L*. *interrogans* serovar Pomona, thereby providing stronger evidence for surface localization and enhancing the efficacy of total protein coverage compared to the use of only a single method. In addition, to reduce contamination of cytoplasmic and periplasmic proteins, we optimized the concentration of biotin and proK to minimize leptospiral membrane disruption during biotin labeling and proteolytic shaving process.

The Sulfo-NHS-SS-Biotin used in this study is a hydrophilic and cell membrane-impermeable reagent containing sulfonate group on the N-hydroxysuccinimide ring that reacts with primary amines (-NH2) on surface proteins of intact cells. After labeling and purification, Western blot revealed that surface proteins were mainly obtained in the biotinylated protein samples (Fig 3). In parallel, the proK shaving was performed to digest the surface-exposed portion of OMPs at the optimal concentration of proK to prevent cell lysis. The proK was used because it is potent, active at a wide pH range, and low peptide bond specificity adjacent to the carboxyl group of aliphatic and aromatic amino acids resulting in a broad range of surface proteins in the cleaved fraction *[9]*. The immunoblotting revealed that the surface proteins were predominantly released into the supernatant fraction (Fig 4). The outer membrane of leptospires are fragile and easily disrupted [42], therefore leptospiral cells were handled as gently as possible during protein preparations to minimize membrane degradation. However, a certain degree of cell lysis was observed by fluorescence viability staining after the experimental process (Figs 3 and 5).

The conventional proteomics approach using two-dimensional gel electrophoresis (2DE) coupled with matrix assisted laser desorption ionization-time of flight mass spectrometry (MALDI-TOF MS) [10,43], mainly identifies abundant proteins but inefficiently identifies highly hydrophobic or membrane proteins. In more recent studies, LC-MS/MS, a high-throughput and high-resolution method, was used to identify leptospiral membrane proteins from the samples of subcellular fractionation using Triton X-114 [44,45]. Moreover, LC-MS/MS-based studies and isotope labeling couple revealed protein abundance of leptospires [46]. In this study, LC-MS/MS-based surface proteomics and label-free quantification with MaxQuant was used to identify SE-OMPs and calculate their abundance profiles. Surface biotinylation and proK shaving identified a total of 1,001 proteins and 238 proteins, respectively (Fig 6). The difference of results obtained from these two methods was explained by the fact that proK could not cleave all leptospiral surface proteins, especially those lacking proK cleavage sites [12] and biotin might fail to label the proteins that their lysine residues are not exposed [10]. Therefore, the number of SE-OMPs was likely an approximate value.

The previous surface proteome of *Leptospira* revealed that LipL21, LipL32, and LipL41 were abundant on the cell surface [10]. All three proteins were also identified and ranked in the top 20 abundance list in this study (Tables 1 and 2). However LipL32 was previously confirmed as a subsurface lipoprotein [13], therefore LipL41 was the highest abundant SE-OMP followed by EF-Tu, LipL21, LipL46, LipL71, Loa22, OmpL36. Moreover, we also identified McpA, OmpL1, OmpL32, SdhA, SppA, LIC10314, and LIC12615 as high-abundance SE-OMPs since their abundances were ranked in the top 50 abundance list (S3 Table). These proteins are conserved among pathogenic leptospires, expressed during mammalian infection, and involved in leptospiral pathogenesis [47–52]. For example, the leptospires mutant lacking Loa22 expression was attenuated in animal models [50]. LipL46 and OmpL32 were detected in *Leptospira* residing in tissues of infected animals [51,52]. OmpL36, LipL46, and EF-Tu were able to interact with several host components [47–49]. Previous studies demonstrated the potential vaccine candidates and target proteins for diagnostic tests of high abundance SE-OMPs. For example, LipL21, LipL41, and OmpL1 have been tested as vaccine candidates and certain combinations conferred synergistic effect on protection [53,54], supporting the rationale to use high-abundance SE-OMPs in multi-subunit or chimeric vaccines. Recombinant LipL21, LipL41, and Loa22 specifically reacted with sera from leptospirosis patients and specific antibodies against these abundance proteins could recognize their native forms on leptospiral cells [55,56], implying the beneficial application of high-abundance SE-OMPs in diagnosis of leptospirosis.

Reverse vaccinology was previously used for screening leptospiral OMPs and SE-OMPs as new vaccine candidates [34,35]. For example, reverse and three-dimensional structural vaccinology predicting conserved βb transmembrane proteins and OM lipoproteins was employed to select the surface-related vaccine candidates [34]. βb-OMPs and OM lipoproteins are major types of membrane proteins that contain SE-region on the cell surface of Gram-negative bacteria [57,58]. Of all uncharacterized proteins listed in the top 50 abundance, only LIC10411 (LIP3228 ortholog in serovar Pomona) contained the predicted property of βb-OMP and shared overlapping in both sample groups. Moreover, it is a conserved and abundance protein in pathogenic serovars [59]. Therefore, it is a promising novel vaccine candidate. However, other characteristics of good vaccine candidates such as *in vivo* expression, immunogenicity, and role in pathogenesis should be further evaluated.

Although low-passage leptospires were used to avoid the loss of virulence or the change of their protein expression after high-passage *in vitro* culture as previously reported [60,61], we might not detect SE-OMPs that were not expressed or were extremely low abundant under *in vitro* culture conditions. In addition, only the whole *L*. *interrogans* serovar Copenhageni Fiocruz L1-130 protein database was available for searching matched mass spectra of proteins, unique proteins in our *L*. *interrogans* serovar Pomona might not be identified. Despite the precautions taken to avoid membrane disruption, a small degree of cell lysis was inevitable and known cytoplasmic proteins and known periplasmic proteins were detected in the surface protein-enriched fractions. In addition, some proteins were named by annotation that might mislead as cytoplasmic proteins, for example, EF-Tu was already confirmed as a surface-exposed outer membrane protein of *Leptospira* [49]. Due to the high sensitivity of LC-MS/MS, even trace amount of proteins contaminated from undesired compartments might be detected, as shown by detection of FlaA1 in the LC-MS/MS results but not on the Western blot. Moreover, some proteins might have multiple subcellular localizations, for example, chaperone proteins including GroEL, DnaK and ClpB [62].

In conclusion, our results demonstrated that the complementary strategy of surface biotinylation and proteinase K shaving followed by LC-MS/MS with label-free quantification was useful to expand the repertoire of surface proteins and the abundance profile of virulent *L*. *interrogans* serovar Pomona. We identified several high-abundance SE-OMPs including EF-Tu, LipL21, LipL41, LipL46, LipL71, Loa22, McpA, OmpL1, OmpL32, OmpL36, SdhA, SppA, LIC10314, and LIC12615. Moreover, we reported the *in silico*-based characterization of LIC10411(LIP3228 ortholog) to be a putative SE-OMP. However, its subcellular localization should be confirmed. Leptospiral surface proteome obtained from this study is useful for further investigation of novel virulence factors of pathogenic leptospires and serves as new targets for vaccine development as well as diagnostic tests for leptospirosis.

## Supporting information

S1 Fig Band intensity of biotinylated proteins calculated by ImageJ. (TIF)

S2 Fig Band intensity of protein fractions calculated by ImageJ. (TIF)

S1 Table Known surface-exposed outer membrane proteins obtained by surface biotinylation and surface shaving. (DOCX)

S2 Table Predicted surface-exposed outer membrane proteins obtained by surface biotinylation and surface shaving. (DOCX)

S3 Table The most 50 abundant proteins obtained by surface biotinylation and surface shaving. (DOCX)

S4 Table Bioinformatics tools used to predict βb-OMP and OM lipoproteins as SE-OMPs. (DOCX)

S1 Appendix All identified proteins and their LFQ intensities. (XLSX)

## References

[1] Bharti AR, Nally JE, Ricaldi JN, Matthias MA, Diaz MM, Lovett MA, et al. Leptospirosis: a zoonotic disease of global importance. Lancet Infect Dis. 2003;3(12):757–71. Epub 2003/12/04. doi: 10.1016/s1473-3099(03)00830-2 . 14652202

[2] Adler B, de la Pena Moctezuma A. *Leptospira* and leptospirosis. Vet Microbiol. 2010;140(3–4):287–96. Epub 2009/04/07. doi: 10.1016/j.vetmic.2009.03.012 . 19345023

[3] Costa F, Hagan JE, Calcagno J, Kane M, Torgerson P, Martinez-Silveira MS, et al. Global Morbidity and Mortality of Leptospirosis: A Systematic Review. PLoS Negl Trop Dis. 2015;9(9):e0003898. Epub 2015/09/18. doi: 10.1371/journal.pntd.0003898 ; PubMed Central PMCID: PMC4574773. 26379143 PMC4574773

[4] Chiriboga J, Barragan V, Arroyo G, Sosa A, Birdsell DN, Espana K, et al. High Prevalence of Intermediate Leptospira spp. DNA in Febrile Humans from Urban and Rural Ecuador. Emerg Infect Dis. 2015;21(12):2141–7. Epub 2015/11/20. doi: 10.3201/eid2112.140659 ; PubMed Central PMCID: PMC4672404. 26583534 PMC4672404

[5] Pinne M, Matsunaga J, Haake DA. Leptospiral outer membrane protein microarray, a novel approach to identification of host ligand-binding proteins. J Bacteriol. 2012;194(22):6074–87. Epub 2012/09/11. doi: 10.1128/JB.01119-12 ; PubMed Central PMCID: PMC3486348. 22961849 PMC3486348

[6] Vieira ML, Fernandes LG, Domingos RF, Oliveira R, Siqueira GH, Souza NM, et al. Leptospiral extracellular matrix adhesins as mediators of pathogen-host interactions. FEMS Microbiol Lett. 2014;352(2):129–39. Epub 2013/12/03. doi: 10.1111/1574-6968.12349 . 24289724

[7] Grandi G. Bacterial surface proteins and vaccines. F1000 Biol Rep. 2010;2. Epub 2010/10/16. doi: 10.3410/B2-2 ; PubMed Central PMCID: PMC2950030. 20948798 PMC2950030

[8] Cordwell SJ. Technologies for bacterial surface proteomics. Curr Opin Microbiol. 2006;9(3):320–9. Epub 2006/05/09. doi: 10.1016/j.mib.2006.04.008 . 16679049

[9] Olaya-Abril A, Jimenez-Munguia I, Gomez-Gascon L, Rodriguez-Ortega MJ. Surfomics: shaving live organisms for a fast proteomic identification of surface proteins. J Proteomics. 2014;97:164–76. Epub 2013/04/30. doi: 10.1016/j.jprot.2013.03.035 . 23624344

[10] Cullen PA, Xu X, Matsunaga J, Sanchez Y, Ko AI, Haake DA, et al. Surfaceome of *Leptospira* spp. Infect Immun. 2005;73(8):4853–63. Epub 2005/07/26. doi: 10.1128/IAI.73.8.4853-4863.2005 ; PubMed Central PMCID: PMC1201201. 16040999 PMC1201201

[11] Cullen PA, Haake DA, Bulach DM, Zuerner RL, Adler B. LipL21 is a novel surface-exposed lipoprotein of pathogenic *Leptospira* species. Infect Immun. 2003;71(5):2414–21. Epub 2003/04/22. doi: 10.1128/IAI.71.5.2414-2421.2003 ; PubMed Central PMCID: PMC153295. 12704111 PMC153295

[12] Pinne M, Haake DA. A comprehensive approach to identification of surface-exposed, outer membrane-spanning proteins of *Leptospira interrogans*. PLoS One. 2009;4(6):e6071. Epub 2009/06/30. doi: 10.1371/journal.pone.0006071 ; PubMed Central PMCID: PMC2698987. 19562037 PMC2698987

[13] Pinne M, Haake DA. LipL32 Is a Subsurface Lipoprotein of Leptospira interrogans: presentation of new data and reevaluation of previous studies. PLoS One. 2013;8(1):e51025. Epub 2013/01/17. doi: 10.1371/journal.pone.0051025 ; PubMed Central PMCID: PMC3544172. 23323152 PMC3544172

[14] Figueredo JM, Siqueira GH, de Souza GO, Heinemann MB, Vasconcellos SA, Chapola EGB, et al. Characterization of two new putative adhesins of *Leptospira interrogans*. Microbiology (Reading). 2017;163(1):37–51. Epub 2017/02/16. doi: 10.1099/mic.0.000411 . 28198346

[15] Tyanova S, Temu T, Cox J. The MaxQuant computational platform for mass spectrometry-based shotgun proteomics. Nat Protoc. 2016;11(12):2301–19. Epub 2016/11/04. doi: 10.1038/nprot.2016.136 . 27809316

[16] Techawiwattanaboon T, Barnier-Quer C, Palaga T, Jacquet A, Collin N, Sangjun N, et al. Reduced Renal Colonization and Enhanced Protection by Leptospiral Factor H Binding Proteins as a Multisubunit Vaccine Against Leptospirosis in Hamsters. Vaccines (Basel). 2019;7(3). Epub 2019/08/25. doi: 10.3390/vaccines7030095 ; PubMed Central PMCID: PMC6789851. 31443566 PMC6789851

[17] Zuerner RL. Laboratory maintenance of pathogenic *Leptospira*. Curr Protoc Microbiol. 2005;Chapter 12:Unit 12E 1. Epub 2008/09/05. doi: 10.1002/9780471729259.mc12e01s00 . 18770554

[18] Litovchick L. Preparing Protein Solutions for Immunoblotting. Cold Spring Harbor Protocols. 2018;2018(7):pdb.prot098418. doi: 10.1101/pdb.prot098418 29967277

[19] Chou CL, Hwang G, Hageman DJ, Han L, Agrawal P, Pisitkun T, et al. Identification of UT-A1- and AQP2-interacting proteins in rat inner medullary collecting duct. Am J Physiol Cell Physiol. 2018;314(1):C99–C117. Epub 2017/10/20. doi: 10.1152/ajpcell.00082.2017 ; PubMed Central PMCID: PMC5866378. 29046292 PMC5866378

[20] Yu NY, Wagner JR, Laird MR, Melli G, Rey S, Lo R, et al. PSORTb 3.0: improved protein subcellular localization prediction with refined localization subcategories and predictive capabilities for all prokaryotes. Bioinformatics. 2010;26(13):1608–15. Epub 2010/05/18. doi: 10.1093/bioinformatics/btq249 ; PubMed Central PMCID: PMC2887053. 20472543 PMC2887053

[21] Yu CS, Lin CJ, Hwang JK. Predicting subcellular localization of proteins for Gram-negative bacteria by support vector machines based on n-peptide compositions. Protein Sci. 2004;13(5):1402–6. Epub 2004/04/21. doi: 10.1110/ps.03479604 ; PubMed Central PMCID: PMC2286765. 15096640 PMC2286765

[22] Shen HB, Chou KC. Gneg-mPLoc: a top-down strategy to enhance the quality of predicting subcellular localization of Gram-negative bacterial proteins. J Theor Biol. 2010;264(2):326–33. Epub 2010/01/23. doi: 10.1016/j.jtbi.2010.01.018 . 20093124

[23] Imai K, Asakawa N, Tsuji T, Akazawa F, Ino A, Sonoyama M, et al. SOSUI-GramN: high performance prediction for sub-cellular localization of proteins in gram-negative bacteria. Bioinformation. 2008;2(9):417–21. Epub 2008/09/17. doi: 10.6026/97320630002417 ; PubMed Central PMCID: PMC2533062. 18795116 PMC2533062

[24] Rahman O, Cummings SP, Harrington DJ, Sutcliffe IC. Methods for the bioinformatic identification of bacterial lipoproteins encoded in the genomes of Gram-positive bacteria. World Journal of Microbiology and Biotechnology. 2008;24(11):2377. doi: 10.1007/s11274-008-9795-2

[25] Petersen TN, Brunak S, von Heijne G, Nielsen H. SignalP 4.0: discriminating signal peptides from transmembrane regions. Nat Methods. 2011;8(10):785–6. Epub 2011/10/01. doi: 10.1038/nmeth.1701 . 21959131

[26] Chou KC, Shen HB. Signal-CF: a subsite-coupled and window-fusing approach for predicting signal peptides. Biochem Biophys Res Commun. 2007;357(3):633–40. Epub 2007/04/17. doi: 10.1016/j.bbrc.2007.03.162 . 17434148

[27] Hiller K, Grote A, Scheer M, Munch R, Jahn D. PrediSi: prediction of signal peptides and their cleavage positions. Nucleic Acids Res. 2004;32(Web Server issue):W375–9. Epub 2004/06/25. doi: 10.1093/nar/gkh378 ; PubMed Central PMCID: PMC441516. 15215414 PMC441516

[28] Krogh A, Larsson B, von Heijne G, Sonnhammer EL. Predicting transmembrane protein topology with a hidden Markov model: application to complete genomes. J Mol Biol. 2001;305(3):567–80. Epub 2001/01/12. doi: 10.1006/jmbi.2000.4315 . 11152613

[29] Kall L, Krogh A, Sonnhammer EL. A combined transmembrane topology and signal peptide prediction method. J Mol Biol. 2004;338(5):1027–36. Epub 2004/04/28. doi: 10.1016/j.jmb.2004.03.016 . 15111065

[30] Dobson L, Remenyi I, Tusnady GE. CCTOP: a Consensus Constrained TOPology prediction web server. Nucleic Acids Res. 2015;43(W1):W408–12. Epub 2015/05/07. doi: 10.1093/nar/gkv451 ; PubMed Central PMCID: PMC4489262. 25943549 PMC4489262

[31] Remmert M, Linke D, Lupas AN, Soding J. HHomp—prediction and classification of outer membrane proteins. Nucleic Acids Res. 2009;37(Web Server issue):W446–51. Epub 2009/05/12. doi: 10.1093/nar/gkp325 ; PubMed Central PMCID: PMC2703889. 19429691 PMC2703889

[32] Bagos PG, Liakopoulos TD, Spyropoulos IC, Hamodrakas SJ. PRED-TMBB: a web server for predicting the topology of beta-barrel outer membrane proteins. Nucleic Acids Res. 2004;32(Web Server issue):W400–4. Epub 2004/06/25. doi: 10.1093/nar/gkh417 ; PubMed Central PMCID: PMC441555. 15215419 PMC441555

[33] Ou YY, Gromiha MM, Chen SA, Suwa M. TMBETADISC-RBF: Discrimination of beta-barrel membrane proteins using RBF networks and PSSM profiles. Comput Biol Chem. 2008;32(3):227–31. Epub 2008/04/25. doi: 10.1016/j.compbiolchem.2008.03.002 . 18434251

[34] Grassmann AA, Kremer FS, Dos Santos JC, Souza JD, Pinto LDS, McBride AJA. Discovery of Novel Leptospirosis Vaccine Candidates Using Reverse and Structural Vaccinology. Front Immunol. 2017;8:463. Epub 2017/05/13. doi: 10.3389/fimmu.2017.00463 ; PubMed Central PMCID: PMC5406399. 28496441 PMC5406399

[35] Zeng L, Wang D, Hu N, Zhu Q, Chen K, Dong K, et al. A Novel Pan-Genome Reverse Vaccinology Approach Employing a Negative-Selection Strategy for Screening Surface-Exposed Antigens against leptospirosis. Front Microbiol. 2017;8:396. Epub 2017/03/30. doi: 10.3389/fmicb.2017.00396 ; PubMed Central PMCID: PMC5348505. 28352257 PMC5348505

[36] Pinne M, Haake D. Immuno-fluorescence assay of leptospiral surface-exposed proteins. J Vis Exp. 2011;(53). Epub 2011/07/14. doi: 10.3791/2805 ; PubMed Central PMCID: PMC3196178. 21750491 PMC3196178

[37] Haake DA, Walker EM, Blanco DR, Bolin CA, Miller MN, Lovett MA. Changes in the surface of *Leptospira interrogans se*rovar grippotyphosa during in vitro cultivation. Infect Immun. 1991;59(3):1131–40. Epub 1991/03/01. doi: 10.1128/iai.59.3.1131-1140.1991 ; PubMed Central PMCID: PMC258378. 1997416 PMC258378

[38] Parveen N, Leong JM. Identification of a candidate glycosaminoglycan-binding adhesin of the Lyme disease spirochete *Borrelia burgdorferi*. Mol Microbiol. 2000;35(5):1220–34. Epub 2000/03/11. doi: 10.1046/j.1365-2958.2000.01792.x . 10712702

[39] Sabarth N, Lamer S, Zimny-Arndt U, Jungblut PR, Meyer TF, Bumann D. Identification of surface proteins of *Helicobacter pylori* by selective biotinylation, affinity purification, and two-dimensional gel electrophoresis. J Biol Chem. 2002;277(31):27896–902. Epub 2002/05/25. doi: 10.1074/jbc.M204473200 . 12023975

[40] Olaya-Abril A, Gomez-Gascon L, Jimenez-Munguia I, Obando I, Rodriguez-Ortega MJ. Another turn of the screw in shaving Gram-positive bacteria: Optimization of proteomics surface protein identification in *Streptococcus pneumoniae*. J Proteomics. 2012;75(12):3733–46. Epub 2012/05/12. doi: 10.1016/j.jprot.2012.04.037 . 22575384

[41] Solis N, Larsen MR, Cordwell SJ. Improved accuracy of cell surface shaving proteomics in Staphylococcus aureus using a false-positive control. Proteomics. 2010;10(10):2037–49. Epub 2010/03/11. doi: 10.1002/pmic.200900564 . 20217865

[42] Rey S, Gardy JL, Brinkman FS. Assessing the precision of high-throughput computational and laboratory approaches for the genome-wide identification of protein subcellular localization in bacteria. BMC Genomics. 2005;6:162. Epub 2005/11/18. doi: 10.1186/1471-2164-6-162 ; PubMed Central PMCID: PMC1314894. 16288665 PMC1314894

[43] Cullen PA, Cordwell SJ, Bulach DM, Haake DA, Adler B. Global analysis of outer membrane proteins from *Leptospira interrogans* serovar Lai. Infect Immun. 2002;70(5):2311–8. Epub 2002/04/16. doi: 10.1128/IAI.70.5.2311-2318.2002 ; PubMed Central PMCID: PMC127947. 11953365 PMC127947

[44] Thoduvayil S, Dhandapani G, Brahma R, Devasahayam Arokia Balaya R, Mangalaparthi KK, Patel K, et al. Triton X-114 Fractionated Subcellular Proteome of *Leptospira interrogans* Shows Selective Enrichment of Pathogenic and Outer Membrane Proteins in the Detergent Fraction. Proteomics. 2020;20(19–20):e2000170. Epub 2020/08/28. doi: 10.1002/pmic.202000170 . 32846045

[45] Lo M, Cordwell SJ, Bulach DM, Adler B. Comparative transcriptional and translational analysis of leptospiral outer membrane protein expression in response to temperature. PLoS Negl Trop Dis. 2009;3(12):e560. Epub 2009/12/10. doi: 10.1371/journal.pntd.0000560 ; PubMed Central PMCID: PMC2780356. 19997626 PMC2780356

[46] Malmstrom J, Beck M, Schmidt A, Lange V, Deutsch EW, Aebersold R. Proteome-wide cellular protein concentrations of the human pathogen *Leptospira interrogans*. Nature. 2009;460(7256):762–5. Epub 2009/07/17. doi: 10.1038/nature08184 ; PubMed Central PMCID: PMC2723184. 19606093 PMC2723184

[47] Pinne M, Choy HA, Haake DA. The OmpL37 surface-exposed protein is expressed by pathogenic *Leptospira* during infection and binds skin and vascular elastin. PLoS Negl Trop Dis. 2010;4(9):e815. Epub 2010/09/17. doi: 10.1371/journal.pntd.0000815 ; PubMed Central PMCID: PMC2935396. 20844573 PMC2935396

[48] Santos JV, Pereira PRM, Fernandes LGV, Siqueira GH, de Souza GO, Souza Filho A, et al. Binding of human plasminogen by the lipoprotein LipL46 of *Leptospira interrogans*. Mol Cell Probes. 2018;37:12–21. Epub 2017/11/08. doi: 10.1016/j.mcp.2017.10.004 . 29108931

[49] Wolff DG, Castiblanco-Valencia MM, Abe CM, Monaris D, Morais ZM, Souza GO, et al. Interaction of Leptospira elongation factor Tu with plasminogen and complement factor H: a metabolic leptospiral protein with moonlighting activities. PLoS One. 2013;8(11):e81818. Epub 2013/12/07. doi: 10.1371/journal.pone.0081818 ; PubMed Central PMCID: PMC3842364. 24312361 PMC3842364

[50] Ristow P, Bourhy P, da Cruz McBride FW, Figueira CP, Huerre M, Ave P, et al. The OmpA-like protein Loa22 is essential for leptospiral virulence. PLoS Pathog. 2007;3(7):e97. Epub 2007/07/17. doi: 10.1371/journal.ppat.0030097 ; PubMed Central PMCID: PMC1914066. 17630832 PMC1914066

[51] Matsunaga J, Werneid K, Zuerner RL, Frank A, Haake DA. LipL46 is a novel surface-exposed lipoprotein expressed during leptospiral dissemination in the mammalian host. Microbiology (Reading). 2006;152(Pt 12):3777–86. Epub 2006/12/13. doi: 10.1099/mic.0.29162-0 ; PubMed Central PMCID: PMC2667200. 17159228 PMC2667200

[52] Eshghi A, Pinne M, Haake DA, Zuerner RL, Frank A, Cameron CE. Methylation and in vivo expression of the surface-exposed *Leptospira interrogans* outer-membrane protein OmpL32. Microbiology (Reading). 2012;158(Pt 3):622–35. Epub 2011/12/17. doi: 10.1099/mic.0.054767-0 ; PubMed Central PMCID: PMC3352116. 22174381 PMC3352116

[53] Haake DA, Mazel MK, McCoy AM, Milward F, Chao G, Matsunaga J, et al. Leptospiral outer membrane proteins OmpL1 and LipL41 exhibit synergistic immunoprotection. Infect Immun. 1999;67(12):6572–82. Epub 1999/11/24. doi: 10.1128/IAI.67.12.6572-6582.1999 ; PubMed Central PMCID: PMC97069. 10569777 PMC97069

[54] Luo D, Xue F, Ojcius DM, Zhao J, Mao Y, Li L, et al. Protein typing of major outer membrane lipoproteins from Chinese pathogenic Leptospira spp. and characterization of their immunogenicity. Vaccine. 2009;28(1):243–55. Epub 2009/10/03. doi: 10.1016/j.vaccine.2009.09.089 . 19796723

[55] Chalayon P, Chanket P, Boonchawalit T, Chattanadee S, Srimanote P, Kalambaheti T. Leptospirosis serodiagnosis by ELISA based on recombinant outer membrane protein. Trans R Soc Trop Med Hyg. 2011;105(5):289–97. Epub 2011/03/01. doi: 10.1016/j.trstmh.2011.01.008 . 21353274

[56] Seenichamy A, Bahaman AR, Mutalib AR, Khairani-Bejo S. Production and characterization of a polyclonal antibody of anti-rLipL21-IgG against leptospira for early detection of acute leptospirosis. Biomed Res Int. 2014;2014:592858. Epub 2014/05/27. doi: 10.1155/2014/592858 ; PubMed Central PMCID: PMC4016889. 24860824 PMC4016889

[57] Schulz GE. The structure of bacterial outer membrane proteins. Biochim Biophys Acta. 2002;1565(2):308–17. Epub 2002/11/01. doi: 10.1016/s0005-2736(02)00577-1 . 12409203

[58] Wilson MM, Bernstein HD. Surface-Exposed Lipoproteins: An Emerging Secretion Phenomenon in Gram-Negative Bacteria. Trends Microbiol. 2016;24(3):198–208. Epub 2015/12/30. doi: 10.1016/j.tim.2015.11.006 . 26711681 PMC9373711

[59] Humphryes PC, Weeks ME, Gielbert A, Thomson G, Coldham NG. Analysis of multiple Leptospira interrogans serovar Canicola vaccine proteomes and identification of LipL32 as a biomarker for potency. Clin Vaccine Immunol. 2012;19(4):587–93. Epub 2012/02/11. doi: 10.1128/CVI.05622-11 ; PubMed Central PMCID: PMC3318284. 22323560 PMC3318284

[60] Vieira ML, Pimenta DC, de Morais ZM, Vasconcellos SA, Nascimento AL. Proteome analysis of *Leptospira interrogans* virulent strain. Open Microbiol J. 2009;3:69–74. Epub 2009/07/11. doi: 10.2174/1874285800903010069 ; PubMed Central PMCID: PMC2698427. 19590580 PMC2698427

[61] Matsunaga J, Barocchi MA, Croda J, Young TA, Sanchez Y, Siqueira I, et al. Pathogenic *Leptospira* species express surface-exposed proteins belonging to the bacterial immunoglobulin superfamily. Mol Microbiol. 2003;49(4):929–45. Epub 2003/08/02. doi: 10.1046/j.1365-2958.2003.03619.x ; PubMed Central PMCID: PMC1237129. 12890019 PMC1237129

[62] Wang W, Jeffery CJ. An analysis of surface proteomics results reveals novel candidates for intracellular/surface moonlighting proteins in bacteria. Mol Biosyst. 2016;12(5):1420–31. Epub 2016/03/05. doi: 10.1039/c5mb00550g . 26938107

