# Supplementary figures and images for: Surface proteomics and label-free quantification of Leptospira interrogans serovar Pomona
Source: PLoS Negl Trop Dis. 2021 Nov 29;15(11):e0009983. doi: 10.1371/journal.pntd.0009983 (PMC8659334; doi:10.1371/journal.pntd.0009983)

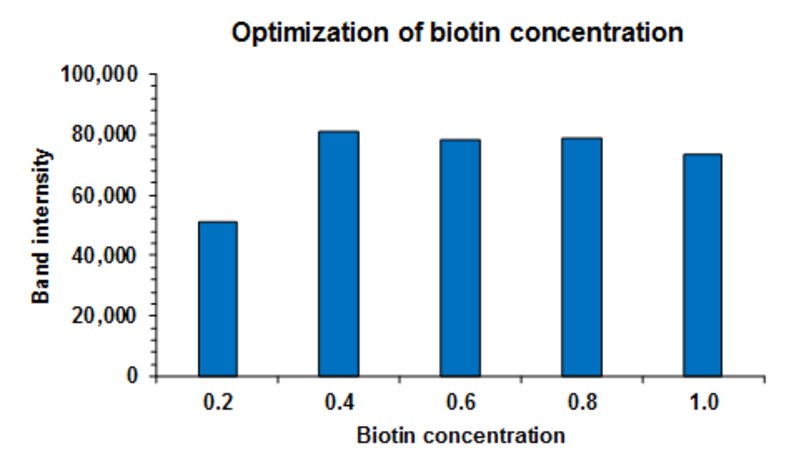

Supplement: S1 Fig — (TIF) [file pntd.0009983.s001.tif]

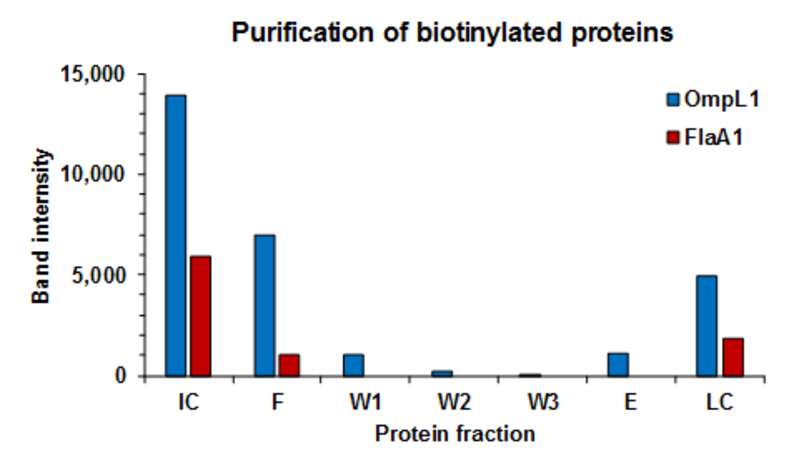

Supplement: S2 Fig — (TIF) [file pntd.0009983.s002.tif]
