# Supplementary material for: Surface proteomics and label-free quantification of Leptospira interrogans serovar Pomona
Source: PLoS Negl Trop Dis. 2021 Nov 29;15(11):e0009983. doi: 10.1371/journal.pntd.0009983 (PMC8659334; doi:10.1371/journal.pntd.0009983)
Supplement: S1 Table — (DOCX) [file pntd.0009983.s003.docx]

**S1 Table** Known surface-exposed outer membrane proteins obtained by surface biotinylation and surface shaving.

| Gene ID | Gene name | Protein ID | Protein function | Abundance ranking | | Reference |
| --- | --- | --- | --- | --- | --- | --- |
|  |  |  |  | Surface biotinylation^a^ | Surface shaving^b^ |  |
| LIC10009 | *lp25* | Q72WC8 | Putative lipoprotein | 452 | ND | [1] |
| **LIC10011** | ***lipL21*** | **Q72WC6** | **LipL21** | **33** | **13** | **[2]** |
| LIC10054 | *mpL36* | Q72W83 | Probable endolytic peptidoglycan transglycosylase | 990 | ND | [3] |
| LIC10091 | *lipL40* | Q72W49 | Putative lipoprotein | ND | ND | [3] |
| **LIC10191** | ***loa22*** | **Q72VV5** | **Peptidoglycan associated cytoplasmic membrane protein** | **7** | **76** | **[4, 5]** |
| LIC10258 | *ompA, lsa66* | Q72VN8 | OmpA-like domain-containing protein | ND | ND | [6] |
| **LIC10314** | ***lsa63*** | **Q72VI3** | **Uncharacterized protein** | **139** | **48** | **[3, 7]** |
| LIC10368 | *lsa21* | Q72VD2 | Putative lipoprotein | ND | ND | [8] |
| LIC10464 | *ligB* | Q72V39 | Ig-like repeat domain protein 3 | ND | ND | [9] |
| LIC10465 | *ligA* | G1UB65 | Ig-like repeat domain protein 1 | 866 | ND | [9] |
| LIC10486 | *pL40* | Q72V17 | Uncharacterized protein | 199 | ND | [10] |
| LIC10494 | *lic10494* | Q72V09 | Putative lipoprotein | ND | ND | [3] |
| LIC10509 | *lic10509* | Q72UZ4 | Putative lipoprotein | ND | ND | [3] |
| LIC10537 | *mfn9* | Q72UX0 | Peptidoglycan associated periplasmic protein | ND | ND | [11] |
| LIC10574 | *omp52* | Q72UT3 | Dihydroorotase | ND | ND | [12] |
| LIC10714 | *mfn2* | Q72UE3 | Outer membrane receptor protein | 555 | ND | [11] |
| LIC10765 | *mpL17* | Q72U96 | Uncharacterized protein | ND | ND | [3, 13] |
| LIC10774 | *lic10774* | Q72U87 | Putative lipoprotein | ND | ND | [14] |
| LIC10793 | *lp49* | Q72U69 | Antigen Lp49 | 676 | ND | [3] |
| LIC10879 | *lsa16* | Q72TY5 | Putative lipoprotein | 71 | ND | [15] |
| LIC10920 | *lsa24.9* | Q72TU6 | Putative lipoprotein | ND | ND | [16] |
| **LIC10973** | ***ompL1*** | **Q72TP4** | **Outer membrane protein** | **22** | **37** | **[17]** |
| LIC11009 | *lsa26* | Q72TK9 | Uncharacterized protein | 208 | ND | [18] |
| LIC11030 | *lic11030* | Q72TJ1 | Putative lipoprotein | ND | ND | [19] |
| LIC11087 | *lsa30* | Q72TD4 | Putative lipoprotein | ND | ND | [20] |
| LIC11122 | *lsa19* | Q72T99 | Putative lipoprotein | 820 | ND | [21] |
| LIC11360 | *lsa23* | Q72SL9 | Putative lipoprotein | 317 | ND | [18] |
| LIC11436 | *mfn7* | Q72SE7 | FecR domain-containing protein | ND | 181 | [11] |
| LIC11458 | *ostA* | Q72SC6 | Outer membrane protein, porin superfamily | 934 | ND | [22] |
| LIC11469 | *lsa20* | Q72SB5 | Uncharacterized protein | 732 | ND | [19] |
| **LIC11570** | ***gspD*** | **Q72S17** | **General secretory pathway protein D** | **200** | **98** | **[22]** |
| LIC11612 | *mfn1* | Q72RX6 | Uncharacterized protein | 792 | ND | [11] |
| LIC11623 | *bamA* | Q72RW5 | Outer membrane protein | ND | ND | [22] |
| LIC11711 | *lic11711* | Q72RN4 | Putative lipoprotein | 748 | ND | [23] |
| **LIC11848** | ***ompL32*** | **Q72RA0** | **Uncharacterized protein** | **39** | **96** | **[24]** |
| **LIC11885** | ***lipL46*** | **Q72R63** | **Putative lipoprotein** | **15** | **19** | **[25]** |
| LIC11947 | *lcpA* | Q72R05 | Putative lipoprotein | 909 | ND | [26] |
| LIC11975 | *lsa36* | Q72QY3 | Outer membrane protein | ND | ND | [18] |
| LIC11996 | *erpY-like* | Q72QY9 | Uncharacterized protein | 327 | ND | [27] |
| LIC12238 | *lic12238* | Q72Q74 | Uncharacterized protein | 977 | ND | [3] |
| LIC12254 | *ompL85* | Q72Q59 | Outer membrane protein | 590 | ND | [22] |
| LIC12263 | *ompL37* | Q72Q50 | Uncharacterized protein | 134 | ND | [17] |
| LIC12287 | *lsa14* | Q72Q27 | Putative lipoprotein | ND | ND | [21] |
| LIC12307 | *tolC* | Q72Q08 | Outer membrane TolC superfamily | ND | ND | [22] |
| LIC12587 | *lic12587* | Q72P88 | Putative lipoprotein | ND | ND | [23] |
| LIC12730 | *lic12730* | Q72NU9 | TPR-REGION domain-containing protein | 832 | ND | [3] |
| **LIC12875** | ***tuf*** | **Q72NF9** | **Elongation factor Tu (EF-Tu)** | **14** | **11** | **[28]** |
| LIC12892 | *lp29* | Q72NE2 | Putative lipoprotein | ND | ND | [3] |
| LIC12895 | *lsa27* | Q72ND9 | Putative lipoprotein | ND | ND | [3, 29] |
| LIC12906 | *lsa24* | Q72NC8 | Putative lipoprotein | ND | ND | [30] |
| LIC12922 | *lic12922* | Q72NB3 | PpiC domain-containing protein | ND | ND | [3] |
| **LIC12966** | ***lipL41*** | **Q72N71** | **LipL41** | **3** | **4** | **[31]** |
| LIC13050 | *ompL47* | Q72MY9 | Uncharacterized protein | 800 | ND | [17] |
| LIC13059 | *lsa25.6* | Q72MY0 | Putative lipoprotein | 291 | ND | [15] |
| LIC13131 | *mpL21* | Q72MR2 | YceI domain-containing protein | ND | ND | [3, 13] |
| LIC13143 | *tlyC* | Q72MQ0 | Hemolysin | 940 | ND | [32] |
| **LIC13166** | ***ompL36*** | **Q72MM7** | **OmpL36** | **8** | **18** | **[17]** |
| LIC13259 | *lic13259* | Q72MC9 | Putative lipoprotein | 474 | ND | [33] |
| LIC13341 | *lic13341* | Q72M50 | Putative lipoprotein | 360 | ND | [34] |
| LIC13491 | *ompL54* | Q72LR2 | Metallopeptidase | 399 | ND | [17] |
| LIC20035 | *lic20035* | Q75G29 | Uncharacterized protein | 894 | ND | [35] |
| LIC20151 | *hbpA* | Q75FN1 | TonB-dependent outer membrane receptor | ND | ND | [36] |
| **LIC20172** | ***lruC*** | **Q75FL0** | **Lipoprotein** | **205** | **70** | **[37, 38]** |

^a^ The ranking was calculated throughout all identified proteins of surface biotinylation, ND means not detected.

^b^ The ranking was calculated throughout all identified proteins of surface proK shaving, ND means not detected.

Bold fonts imply overlapping proteins.

**Reference**

1. Abreu PAE,Seguro AC,Canale D,Silva A,Matos L,Gotti TB, et al. Lp25 membrane protein from pathogenic *Leptospira* spp. is associated with rhabdomyolysis and oliguric acute kidney injury in a guinea pig model of leptospirosis. PLoS Negl Trop Dis. 2017;11(5):e0005615. Epub 2017/05/16. doi: 10.1371/journal.pntd.0005615. PubMed PMID: 28505191; PubMed Central PMCID: PMCPMC5444857.

2. Cullen PA,Haake DA,Bulach DM,Zuerner RL, Adler B. LipL21 is a novel surface-exposed lipoprotein of pathogenic *Leptospira* species. Infect Immun. 2003;71(5):2414-21. Epub 2003/04/22. doi: 10.1128/iai.71.5.2414-2421.2003. PubMed PMID: 12704111; PubMed Central PMCID: PMCPMC153295.

3. Vieira ML,Atzingen MV,Oliveira TR,Oliveira R,Andrade DM,Vasconcellos SA, et al. In vitro identification of novel plasminogen-binding receptors of the pathogen *Leptospira interrogans*. PLoS One. 2010;5(6):e11259. Epub 2010/06/29. doi: 10.1371/journal.pone.0011259. PubMed PMID: 20582320; PubMed Central PMCID: PMCPMC2889836.

4. Koizumi N, Watanabe H. Molecular cloning and characterization of a novel leptospiral lipoprotein with OmpA domain. FEMS Microbiol Lett. 2003;226(2):215-9. Epub 2003/10/14. doi: 10.1016/S0378-1097(03)00619-0. PubMed PMID: 14553914.

5. Ristow P,Bourhy P,da Cruz McBride FW,Figueira CP,Huerre M,Ave P, et al. The OmpA-like protein Loa22 is essential for leptospiral virulence. PLoS Pathog. 2007;3(7):e97. Epub 2007/07/17. doi: 10.1371/journal.ppat.0030097. PubMed PMID: 17630832; PubMed Central PMCID: PMCPMC1914066.

6. Oliveira R,de Morais ZM,Goncales AP,Romero EC,Vasconcellos SA, Nascimento AL. Characterization of novel OmpA-like protein of *Leptospira interrogans* that binds extracellular matrix molecules and plasminogen. PLoS One. 2011;6(7):e21962. Epub 2011/07/15. doi: 10.1371/journal.pone.0021962. PubMed PMID: 21755014; PubMed Central PMCID: PMCPMC3130794.

7. Vieira ML,de Morais ZM,Goncales AP,Romero EC,Vasconcellos SA, Nascimento AL. Lsa63, a newly identified surface protein of *Leptospira interrogans* binds laminin and collagen IV. J Infect. 2010;60(1):52-64. Epub 2009/11/03. doi: 10.1016/j.jinf.2009.10.047. PubMed PMID: 19879894.

8. Atzingen MV,Barbosa AS,De Brito T,Vasconcellos SA,de Morais ZM,Lima DM, et al. Lsa21, a novel leptospiral protein binding adhesive matrix molecules and present during human infection. BMC Microbiol. 2008;8:70. Epub 2008/05/01. doi: 10.1186/1471-2180-8-70. PubMed PMID: 18445272; PubMed Central PMCID: PMCPMC2386478.

9. Matsunaga J,Barocchi MA,Croda J,Young TA,Sanchez Y,Siqueira I, et al. Pathogenic *Leptospira* species express surface-exposed proteins belonging to the bacterial immunoglobulin superfamily. Mol Microbiol. 2003;49(4):929-45. Epub 2003/08/02. doi: 10.1046/j.1365-2958.2003.03619.x. PubMed PMID: 12890019; PubMed Central PMCID: PMCPMC1237129.

10. Zhao W,Chen CY,Zhang XY,Lai WQ,Hu BY,Zhao GP, et al. Molecular characterization of the pL40 protein in *Leptospira interrogans*. Can J Microbiol. 2009;55(6):739-49. Epub 2009/09/22. doi: 10.1139/w09-014. PubMed PMID: 19767845.

11. Pinne M,Matsunaga J, Haake DA. Leptospiral outer membrane protein microarray, a novel approach to identification of host ligand-binding proteins. J Bacteriol. 2012;194(22):6074-87. Epub 2012/09/11. doi: 10.1128/JB.01119-12. PubMed PMID: 22961849; PubMed Central PMCID: PMCPMC3486348.

12. Hsieh WJ,Chang YF,Chen CS, Pan MJ. Omp52 is a growth-phase-regulated outer membrane protein of *Leptospira santarosai* serovar Shermani. FEMS Microbiol Lett. 2005;243(2):339-45. Epub 2005/02/03. doi: 10.1016/j.femsle.2004.12.021. PubMed PMID: 15686833.

13. Oliveira TR,Longhi MT,de Morais ZM,Romero EC,Blanco RM,Kirchgatter K, et al. Evaluation of leptospiral recombinant antigens MPL17 and MPL21 for serological diagnosis of leptospirosis by enzyme-linked immunosorbent assays. Clin Vaccine Immunol. 2008;15(11):1715-22. Epub 2008/09/19. doi: 10.1128/CVI.00214-08. PubMed PMID: 18799647; PubMed Central PMCID: PMCPMC2583518.

14. Passalia FJ,Carvalho E,Heinemann MB,Vieira ML, Nascimento A. The Leptospira interrogans LIC10774 is a multifunctional surface protein that binds calcium and interacts with host components. Microbiol Res. 2020;235:126470. Epub 2020/04/06. doi: 10.1016/j.micres.2020.126470. PubMed PMID: 32247916.

15. Pereira PRM,Fernandes LGV,de Souza GO,Vasconcellos SA,Heinemann MB,Romero EC, et al. Multifunctional and Redundant Roles of *Leptospira interrogans* Proteins in Bacterial-Adhesion and fibrin clotting inhibition. Int J Med Microbiol. 2017;307(6):297-310. Epub 2017/06/11. doi: 10.1016/j.ijmm.2017.05.006. PubMed PMID: 28600123.

16. Karlsson R,Thorell K,Hosseini S,Kenny D,Sihlbom C,Sjoling A, et al. Comparative Analysis of Two *Helicobacter pylori* Strains using Genomics and Mass Spectrometry-Based Proteomics. Front Microbiol. 2016;7:1757. Epub 2016/11/29. doi: 10.3389/fmicb.2016.01757. PubMed PMID: 27891114; PubMed Central PMCID: PMCPMC5104757.

17. Pinne M, Haake DA. A comprehensive approach to identification of surface-exposed, outer membrane-spanning proteins of *Leptospira interrogans*. PLoS One. 2009;4(6):e6071. Epub 2009/06/30. doi: 10.1371/journal.pone.0006071. PubMed PMID: 19562037; PubMed Central PMCID: PMCPMC2698987.

18. Siqueira GH,Atzingen MV,Alves IJ,de Morais ZM,Vasconcellos SA, Nascimento AL. Characterization of three novel adhesins of Leptospira interrogans. Am J Trop Med Hyg. 2013;89(6):1103-16. Epub 2013/08/21. doi: 10.4269/ajtmh.13-0205. PubMed PMID: 23958908; PubMed Central PMCID: PMCPMC3854887.

19. Mendes RS,Von Atzingen M,de Morais ZM,Goncales AP,Serrano SM,Asega AF, et al. The novel leptospiral surface adhesin Lsa20 binds laminin and human plasminogen and is probably expressed during infection. Infect Immun. 2011;79(11):4657-67. Epub 2011/08/17. doi: 10.1128/IAI.05583-11. PubMed PMID: 21844229; PubMed Central PMCID: PMCPMC3257903.

20. Souza NM,Vieira ML,Alves IJ,de Morais ZM,Vasconcellos SA, Nascimento AL. Lsa30, a novel adhesin of *Leptospira interrogans* binds human plasminogen and the complement regulator C4bp. Microb Pathog. 2012;53(3-4):125-34. Epub 2012/06/27. doi: 10.1016/j.micpath.2012.06.001. PubMed PMID: 22732096.

21. Figueredo JM,Siqueira GH,de Souza GO,Heinemann MB,Vasconcellos SA,Chapola EGB, et al. Characterization of two new putative adhesins of *Leptospira interrogans*. Microbiology (Reading). 2017;163(1):37-51. Epub 2017/02/16. doi: 10.1099/mic.0.000411. PubMed PMID: 28198346.

22. Haake DA, Matsunaga J. Leptospira: a spirochaete with a hybrid outer membrane. Mol Microbiol. 2010;77(4):805-14. Epub 2010/07/06. doi: 10.1111/j.1365-2958.2010.07262.x. PubMed PMID: 20598085; PubMed Central PMCID: PMCPMC2976823.

23. Kochi LT,Fernandes LGV,Souza GO,Vasconcellos SA,Heinemann MB,Romero EC, et al. The interaction of two novel putative proteins of *Leptospira interrogans* with E-cadherin, plasminogen and complement components with potential role in bacterial infection. Virulence. 2019;10(1):734-53. Epub 2019/08/20. doi: 10.1080/21505594.2019.1650613. PubMed PMID: 31422744; PubMed Central PMCID: PMCPMC6735628.

24. Eshghi A,Pinne M,Haake DA,Zuerner RL,Frank A, Cameron CE. Methylation and in vivo expression of the surface-exposed *Leptospira interrogans* outer-membrane protein OmpL32. Microbiology (Reading). 2012;158(Pt 3):622-35. Epub 2011/12/17. doi: 10.1099/mic.0.054767-0. PubMed PMID: 22174381; PubMed Central PMCID: PMCPMC3352116.

25. Matsunaga J,Werneid K,Zuerner RL,Frank A, Haake DA. LipL46 is a novel surface-exposed lipoprotein expressed during leptospiral dissemination in the mammalian host. Microbiology (Reading). 2006;152(Pt 12):3777-86. Epub 2006/12/13. doi: 10.1099/mic.0.29162-0. PubMed PMID: 17159228; PubMed Central PMCID: PMCPMC2667200.

26. Barbosa AS,Monaris D,Silva LB,Morais ZM,Vasconcellos SA,Cianciarullo AM, et al. Functional characterization of LcpA, a surface-exposed protein of *Leptospira* spp. that binds the human complement regulator C4BP. Infect Immun. 2010;78(7):3207-16. Epub 2010/04/21. doi: 10.1128/IAI.00279-10. PubMed PMID: 20404075; PubMed Central PMCID: PMCPMC2897400.

27. Ghosh KK,Prakash A,Dhara A,Hussain MS,Shrivastav P,Kumar P, et al. Role of Supramolecule ErpY-Like Lipoprotein of Leptospira in Thrombin-Catalyzed Fibrin Clot Inhibition and Binding to Complement Factors H and I, and Its Diagnostic Potential. Infect Immun. 2019;87(12). Epub 2019/09/25. doi: 10.1128/IAI.00536-19. PubMed PMID: 31548314; PubMed Central PMCID: PMCPMC6867842.

28. Wolff DG,Castiblanco-Valencia MM,Abe CM,Monaris D,Morais ZM,Souza GO, et al. Interaction of Leptospira elongation factor Tu with plasminogen and complement factor H: a metabolic leptospiral protein with moonlighting activities. PLoS One. 2013;8(11):e81818. Epub 2013/12/07. doi: 10.1371/journal.pone.0081818. PubMed PMID: 24312361; PubMed Central PMCID: PMCPMC3842364.

29. Longhi MT,Oliveira TR,Romero EC,Goncales AP,de Morais ZM,Vasconcellos SA, et al. A newly identified protein of *Leptospira interrogans* mediates binding to laminin. J Med Microbiol. 2009;58(Pt 10):1275-82. Epub 2009/06/23. doi: 10.1099/jmm.0.011916-0. PubMed PMID: 19541787.

30. Barbosa AS,Abreu PA,Neves FO,Atzingen MV,Watanabe MM,Vieira ML, et al. A newly identified leptospiral adhesin mediates attachment to laminin. Infect Immun. 2006;74(11):6356-64. Epub 2006/09/07. doi: 10.1128/IAI.00460-06. PubMed PMID: 16954400; PubMed Central PMCID: PMCPMC1695492.

31. Shang ES,Summers TA, Haake DA. Molecular cloning and sequence analysis of the gene encoding LipL41, a surface-exposed lipoprotein of pathogenic *Leptospira* species. Infect Immun. 1996;64(6):2322-30. Epub 1996/06/01. doi: 10.1128/IAI.64.6.2322-2330.1996. PubMed PMID: 8675344; PubMed Central PMCID: PMCPMC174073.

32. Carvalho E,Barbosa AS,Gomez RM,Cianciarullo AM,Hauk P,Abreu PA, et al. Leptospiral TlyC is an extracellular matrix-binding protein and does not present hemolysin activity. FEBS Lett. 2009;583(8):1381-5. Epub 2009/03/31. doi: 10.1016/j.febslet.2009.03.050. PubMed PMID: 19328790.

33. Cavenague MF,Teixeira AF,Filho AS,Souza GO,Vasconcellos SA,Heinemann MB, et al. Characterization of a novel protein of *Leptospira interrogans e*xhibiting plasminogen, vitronectin and complement binding properties. Int J Med Microbiol. 2019;309(2):116-29. Epub 2019/01/15. doi: 10.1016/j.ijmm.2018.12.005. PubMed PMID: 30638770.

34. Ghosh KK,Prakash A,Shrivastav P,Balamurugan V, Kumar M. Evaluation of a novel outer membrane surface-exposed protein, LIC13341 of *Leptospira*, as an adhesin and serodiagnostic candidate marker for leptospirosis. Microbiology (Reading). 2018;164(8):1023-37. Epub 2018/07/04. doi: 10.1099/mic.0.000685. PubMed PMID: 29969088.

35. Ghosh KK,Prakash A,Balamurugan V, Kumar M. Catecholamine-Modulated Novel Surface-Exposed Adhesin LIC20035 of Leptospira spp. Binds Host Extracellular Matrix Components and Is Recognized by the Host during Infection. Appl Environ Microbiol. 2018;84(6). Epub 2017/12/23. doi: 10.1128/AEM.02360-17. PubMed PMID: 29269501; PubMed Central PMCID: PMCPMC5835738.

36. Asuthkar S,Velineni S,Stadlmann J,Altmann F, Sritharan M. Expression and characterization of an iron-regulated hemin-binding protein, HbpA, from *Leptospira interrogans* serovar Lai. Infect Immun. 2007;75(9):4582-91. Epub 2007/06/20. doi: 10.1128/IAI.00324-07. PubMed PMID: 17576761; PubMed Central PMCID: PMCPMC1951163.

37. Verma A,Matsunaga J,Artiushin S,Pinne M,Houwers DJ,Haake DA, et al. Antibodies to a novel leptospiral protein, LruC, in the eye fluids and sera of horses with *Leptospira*-associated uveitis. Clin Vaccine Immunol. 2012;19(3):452-6. Epub 2012/01/13. doi: 10.1128/CVI.05524-11. PubMed PMID: 22237897; PubMed Central PMCID: PMCPMC3294619.

38. Toma C,Murray GL,Nohara T,Mizuyama M,Koizumi N,Adler B, et al. Leptospiral outer membrane protein LMB216 is involved in enhancement of phagocytic uptake by macrophages. Cell Microbiol. 2014;16(9):1366-77. Epub 2014/03/25. doi: 10.1111/cmi.12296. PubMed PMID: 24655538.
