# Supplementary material for: Surface proteomics and label-free quantification of Leptospira interrogans serovar Pomona
Source: PLoS Negl Trop Dis. 2021 Nov 29;15(11):e0009983. doi: 10.1371/journal.pntd.0009983 (PMC8659334; doi:10.1371/journal.pntd.0009983)
Supplement: S2 Table — (DOCX) [file pntd.0009983.s004.docx]

**S2 Table** Predicted surface-exposed outer membrane proteins obtained by surface biotinylation and surface shaving.

| Gene ID | Gene name | Protein ID | Protein function | Abundance ranking | | Reference |
| --- | --- | --- | --- | --- | --- | --- |
|  |  |  |  | Surface biotinylation^a^ | Surface shaving^b^ |  |
| LIC10010 | *lic10010* | Q72WC7 | Uncharacterized protein | ND | ND | [1] |
| **LIC10011** | ***lipL21*** | **Q72WC6** | **LipL21** | **33** | **13** | [1, 2] |
| LIC10012 | *lic10012* | Q72WC5 | Uncharacterized protein | ND | ND | [1] |
| LIC10017 | *lic10017* | Q72WC0 | Uncharacterized protein | ND | ND | [1] |
| LIC10021 | *lic10021* | Q72WB6 | Putative lipoprotein | ND | ND | [3] |
| LIC10024 | *lic10024* | Q72WB3 | Adenylate/guanylate cyclase | ND | ND | [1] |
| LIC10050 | *lic10050* | Q72W87 | Peptidoglycan-associated cytoplasmic membrane protein | ND | ND | [1] |
| LIC10064 | *lic10064* | Q72W73 | DUF2147 domain-containing protein | 637 | ND | [3] |
| LIC10067 | *lic10067* | Q72W70 | IPT/TIG domain-containing protein | ND | ND | [3] |
| LIC10068 | *lic10068* | Q72W69 | Uncharacterized protein | ND | ND | [3] |
| LIC10087 | *lic10087* | Q72W53 | Uncharacterized protein | ND | ND | [1] |
| LIC10105 | *lic10105* | Q72W35 | Uncharacterized protein | 773 | ND | [1] |
| LIC10123 | *lic10123* | Q72W18 | LipL45-like protein | 303 | ND | [1, 3] |
| **LIC10125** | ***lic10125*** | **Q72W16** | **Uncharacterized protein** | **330** | **125** | **[1]** |
| LIC10207 | *lic10207* | Q72VT9 | SCO1/SenC family protein | 698 | ND | [1] |
| LIC10260 | *lic10260* | Q72VN6 | Putative lipoprotein | ND | ND | [3] |
| LIC10271 | *lic10271* | Q72VM6 | Cell wall hydrolase | ND | ND | [1] |
| LIC10280 | *lic10280* | Q72VL7 | Uncharacterized protein | ND | ND | [3] |
| LIC10288 | *queC* | Q72VK9 | 7-cyano-7-deazaguanine synthase | ND | ND | [3] |
| LIC10298 | *flgC* | Q72VJ9 | Flagellar basal-body rod protein FlgC | ND | ND | [3] |
| LIC10302 | *lic10302* | Q72VJ5 | TPR_REGION domain-containing protein | 952 | ND | [1] |
| LIC10307 | *lic10307* | Q72VJ0 | Putative lipoprotein | ND | ND | [1, 3] |
| LIC10313 | *lic10313* | Q72VI4 | Uncharacterized protein | ND | ND | [3] |
| LIC10314 | *lsa63* | Q72VI3 | Uncharacterized protein | 139 | 48 | [1, 4, 5] |
| LIC10318 | *lic10318* | Q72VH9 | Uncharacterized protein | ND | ND | [1] |
| LIC10325 | *hlyX* | Q72VH2 | Hemolysin | 513 | ND | [1] |
| LIC10326 | *lic10326* | Q72VH1 | Uncharacterized protein | ND | ND | [1] |
| LIC10329 | *lic10329* | Q72VG8 | Uncharacterized protein | ND | ND | [1] |
| LIC10346 | *lic10346* | Q72VF1 | Esterase | ND | ND | [1] |
| LIC10359 | *lic10359* | Q72VD9 | Uncharacterized protein | ND | ND | [1, 3] |
| LIC10368 | *lsa21* | Q72VD2 | Putative lipoprotein | ND | ND | [3, 6] |
| LIC10371 | *lic10371* | Q72VC9 | Putative lipoprotein | ND | ND | [1] |
| LIC10377 | *lic10377* | Q72VC4 | Putative lipoprotein | ND | ND | [3] |
| LIC10405 | *lic10405* | Q72V96 | Probable UDP-N-acetylglucosamine--peptide N-acetylglucosaminyltransferase | ND | ND | [1] |
| LIC10407 | *lic10407* | Q72V94 | Uncharacterized protein | ND | ND | [1] |
| LIC10464 | *ligB* | Q72V39 | Ig-like repeat domain protein 3 | ND | ND | [3, 7] |
| LIC10468 | *lic10468* | Q72V35 | Uncharacterized protein | ND | ND | [1] |
| LIC10496 | *lic10496* | Q72V07 | Uncharacterized protein | ND | ND | [1] |
| LIC10520 | *lic10520* | Q72UY3 | Uncharacterized protein | ND | ND | [1, 3] |
| LIC10537 | *lic10537* | Q72UX0 | Peptidoglycan associated periplasmic protein | ND | ND | [1, 8] |
| LIC10539 | *lic10539* | Q72UW8 | Uncharacterized protein | ND | ND | [1] |
| LIC10544 | *lic10544* | Q72UW3 | Outer membrane protein | ND | ND | [1] |
| LIC10578 | *lic10578* | Q72US9 | Uncharacterized protein | 674 | ND | [1] |
| LIC10628 | *lic10628* | Q72UM9 | Putative lipoprotein | 305 | ND | [3] |
| LIC10647 | *lic10647* | Q72UL0 | Uncharacterized protein | ND | ND | [1] |
| LIC10657 | *sphH* | Q72UK0 | Sphingomyelinase C | ND | ND | [1] |
| LIC10683 | *mauL* | Q72UH4 | Methylamine utilization protein | ND | ND | [3] |
| LIC10686 | *lic10686* | Q72UH1 | Putative lipoprotein | ND | ND | [1, 3] |
| LIC10703 | *lic10703* | Q72UF4 | Uncharacterized protein | ND | ND | [1] |
| LIC10711 | *lic10711* | Q72UE6 | Cytoplasmic membrane protein | ND | ND | [1] |
| LIC10712 | *lic10712* | Q72UE5 | Cytochrome c domain-containing protein | ND | 185 | [3] |
| LIC10713 | *lic10713* | Q72UE4 | Putative lipoprotein | ND | ND | [1, 3] |
| LIC10714 | *mfn2* | Q72UE3 | Outer membrane receptor protein | 555 | ND | [1, 3, 8] |
| LIC10715 | *lic10715* | Q72UE2 | Thermolysin | ND | ND | [1] |
| LIC10723 | *fliD* | Q72UD4 | Flagellar hook-associated protein 2 (HAP2) | ND | ND | [3] |
| LIC10760 | *lic10760* | Q72UA1 | YceI domain-containing protein | 379 | ND | [3] |
| LIC10772 | *lic10772* | Q72U89 | Uncharacterized protein | 696 | ND | [1] |
| LIC10778 | *lic10778* | Q72U83 | Uncharacterized protein | ND | ND | [1] |
| LIC10827 | *lic10827* | Q72U37 | Uncharacterized protein | ND | ND | [1] |
| LIC10833 | *lic10833* | Q72U31 | 3-dmu-9_3-mt domain-containing protein | 895 | ND | [3] |
| **LIC10845** | ***lic10845*** | **Q72U19** | **Uncharacterized protein** | **603** | **191** | **[3]** |
| LIC10869 | *lic10869* | Q72TZ5 | Uncharacterized protein | ND | ND | [1] |
| LIC10873 | *lic10873* | Q72TZ1 | MULTIHEME_CYTC domain-containing protein | 359 | ND | [3] |
| LIC10881 | *lic10881* | Q72TY3 | Outer membrane protein, TonB dependent | ND | ND | [1] |
| LIC10896 | *fecA* | Q72TW8 | TonB-dependent outer membrane receptor | ND | ND | [1] |
| LIC10902 | *lic10902* | Q72TW2 | FecR domain-containing protein | ND | ND | [1] |
| LIC10920 | *lsa24.9* | Q72TU6 | Putative lipoprotein | ND | ND | [3, 9] |
| LIC10963 | *lic10963* | Q72TQ4 | Uncharacterized protein | ND | ND | [3] |
| LIC10964 | *phuR* | Q72TQ3 | TonB-dependent outer membrane hemin receptor | ND | ND | [1] |
| LIC10968 | *lic10968* | Q72TP9 | Uncharacterized protein | ND | ND | [1, 3] |
| **LIC10973** | ***ompL1*** | **Q72TP4** | **Outer membrane protein** | **22** | **37** | [3, 10] |
| **LIC10985** | ***lic10985*** | **Q72TN2** | **Uncharacterized protein** | **179** | **100** | **[1]** |
| **LIC11003** | ***lipL71*** | **Q72TL5** | **LipL71** | **32** | **7** | **[1, 3]** |
| LIC11008 | *lic11008* | Q72TL0 | Uncharacterized protein | ND | ND | [1] |
| LIC11022 |  |  |  |  |  | [1] |
| LIC11028 | *lic11028* | Q72TJ3 | Uncharacterized protein | ND | ND | [1] |
| LIC11030 | *lic11030* | Q72TJ1 | Putative lipoprotein | ND | ND | [3, 11] |
| LIC11040 | *lic11040* | Q72TI1 | Hemolysin | ND | ND | [1, 3] |
| LIC11052 | *lic11052* | Q72TG9 | Uncharacterized protein | ND | ND | [1, 3] |
| LIC11058 | *lemA* | Q72TG3 | Putative lipoprotein | ND | ND | [1] |
| LIC11067 | *lic11067* | Q72TF4 | Uncharacterized protein | 935 | ND | [3] |
| LIC11073 | *lic11073* | Q72TE8 | Putative lipoprotein | ND | ND | [3] |
| LIC11074 | *lic11074* | Q72TE7 | Uncharacterized protein | ND | ND | [3] |
| LIC11082 | *lic11082* | Q72TD9 | Cytoplasmic membrane protein | ND | ND | [1] |
| LIC11084 | *lic11084* | Q72TD7 | Uncharacterized protein | ND | ND | [1] |
| LIC11086 | *lic11086* | Q72TD5 | Uncharacterized protein | ND | ND | [1] |
| LIC11087 | *lsa30* | Q72TD4 | Putative lipoprotein | ND | ND | [1, 3, 12] |
| LIC11103 | *lic11103* | Q72TB8 | Alpha/beta hydrolase superfamily | ND | ND | [1, 3] |
| LIC11111 | *htrA2* | Q72TB0 | HtrA2 | ND | ND | [1] |
| LIC11112 | *lic11112* | Q72TA9 | Serine protease | ND | ND | [1, 3] |
| LIC11117 | *malZ* | Q72TA4 | Alpha-glucosidase | ND | ND | [1] |
| LIC11119 | *lic11119* | Q72TA2 | Uncharacterized protein | ND | ND | [1] |
| LIC11121 | *lic11121* | Q72TA0 | Uncharacterized protein | ND | ND | [1] |
| LIC11122 | *lsa19* | Q72T99 | Putative lipoprotein | 820 | ND | [1, 13] |
| LIC11167 | *lic11167* | Q72T54 | Putative lipoprotein | ND | ND | [1] |
| LIC11184 | *lic11184* | Q72T37 | Putative lipoprotein | ND | ND | [3] |
| LIC11186 | *flbC* | Q72T35 | Putative flagellar protein | ND | ND | [3] |
| LIC11187 | *flgD* | Q72T34 | Basal-body rod modification protein FlgD | ND | ND | [3] |
| LIC11188 | *flgE* | Q72T33 | Flagellar hook protein FlgE | 396 | ND | [3] |
| LIC11207 | *lic11207* | Q72T14 | Putative lipoprotein | ND | 164 | [3] |
| LIC11211 | *lic11211* | Q72T11 | Uncharacterized protein | 1235 | ND | [1] |
| LIC11213 | *lic11213* | Q72T09 | PSCyt1 domain-containing protein | ND | ND | [3] |
| LIC11222 | *lic11222* | Q72T00 | TPR_REGION domain-containing protein | ND | ND | [1] |
| LIC11228 | *lic11228* | Q72SZ4 | Uncharacterized protein | ND | ND | [1, 3] |
| LIC11254 | *lic11254* | Q72SW8 | Putative lipoprotein | ND | ND | [3] |
| LIC11259 | *mreC* | Q72SW3 | Cell shape protein MreC | 525 | ND | [1, 3] |
| LIC11268 | *lic11268* | Q72SV4 | Alginate_exp domain-containing protein | 841 | ND | [1, 3] |
| LIC11270 | *lic11270* | Q72SV2 | Uncharacterized protein | ND | ND | [1, 3] |
| LIC11271 | *lic11271* | Q72SV1 | Uncharacterized protein | ND | ND | [3] |
| LIC11320 | *lic11320* | Q72SQ5 | ATP-cone domain-containing protein | 486 | ND | [3] |
| LIC11327 | *flgI* | Q72SP8 | Flagellar P-ring protein | 260 | ND | [1] |
| LIC11330 |  |  |  |  |  | [1] |
| LIC11334 | *lic11334* | Q72SP3 | Uncharacterized protein | 145 | ND | [1] |
| LIC11345 | *lic11345* | Q72SN4 | Ferrichrome-iron receptor | ND | ND | [1, 3] |
| LIC11358 | *lic11358* | Q72SM1 | Uncharacterized protein | ND | ND | [1] |
| LIC11366 | *lic11366* | Q72SL3 | Uncharacterized protein | 941 | ND | [3] |
| LIC11370 | *fliN* | Q72SK9 | Flagellar motor switch protein FliN | 480 | ND | [3] |
| LIC11371 | *fliO* | Q72SK8 | Flagellar protein | 722 | ND | [1] |
| LIC11382 | *lic11382* | Q72SJ9 | Uncharacterized protein | ND | ND | [3] |
| LIC11388 | *lic11388* | Q72SJ4 | Uncharacterized protein | ND | ND | [3] |
| LIC11424 | *Fkp* | Q72SF9 | Peptidyl-prolyl cis-trans isomerase | 583 | ND | [3] |
| LIC11435 | *lic11435* | Q72SE8 | DUF5683 domain-containing protein | ND | ND | [1, 3] |
| LIC11436 | *mfn7* | Q72SE7 | FecR domain-containing protein | ND | 181 | [1, 8] |
| LIC11458 | *ostA* | Q72SC6 | Outer membrane protein, porin superfamily | 934 | ND | [1, 14] |
| LIC11466 | *lic11466* | Q72SB8 | Uncharacterized protein | ND | ND | [1] |
| LIC11467 | *lic11467* | Q72SB7 | Regulator of chromosome condensation | ND | ND | [3] |
| LIC11468 | *lic11468* | Q72SB6 | Uncharacterized protein | 937 | ND | [1] |
| LIC11486 | *lic11486* | Q72S98 | Uncharacterized protein | 779 | ND | [1] |
| LIC11489 | *lic11489* | Q72S95 | Uncharacterized protein | 124 | ND | [1] |
| LIC11493 | *gldG* | Q72S91 | GldG | ND | ND | [1] |
| LIC11506 | *lic11506* | Q72S78 | Outer membrane protein | ND | ND | [1] |
| LIC11523 | *mcp* | Q72S63 | Chemotaxis protein | ND | ND | [1] |
| LIC11553 | *lic11553* | Q72S33 | Serine/threonine kinase | 215 | ND | [1] |
| LIC11568 | *lic11568* | Q72S19 | Membrane peptidase | ND | ND | [1, 3] |
| LIC11569 | *lic11569* | Q72S18 | Uncharacterized protein | ND | ND | [1] |
| **LIC11570** | ***gspD*** | **Q72S17** | **General secretory pathway protein D** | **200** | **98** | [1, 14] |
| LIC11616 | *rfaD* | Q72RX2 | ADP-L-glycero-D-mannoheptose-6-epimerase | 495 | ND | [1] |
| LIC11623 | *bamA* | Q72RW5 | Outer membrane protein | ND | ND | [1, 14] |
| LIC11625 | *lic11625* | Q72RW3 | Uncharacterized protein | 426 | ND | [3] |
| LIC11628 | *lic11628* | Q72RW0 | Uncharacterized protein | ND | ND | [1] |
| LIC11665 | *lic11665* | Q72RS5 | Uncharacterized protein | ND | ND | [3] |
| LIC11711 | *lic11711* | Q72RN4 | Putative lipoprotein | 748 | ND | [1, 15] |
| LIC11739 | *lic11739* | Q72RK6 | Uncharacterized protein | ND | ND | [3] |
| LIC11754 | *lic11754* | Q72RJ1 | Lipoprotein with phospholipase D domain | ND | ND | [1] |
| LIC11755 | *lic11755* | Q72RJ0 | Uncharacterized protein | ND | ND | [1] |
| **LIC11793** | ***sppA*** | **Q72RF2** | **Signal peptide peptidase** | **35** | **93** | **[1]** |
| LIC11813 | *ggt* | Q72RD3 | Gamma-glutamyltranspeptidase | 271 | ND | [1, 3] |
| LIC11823 | *lic11823* | Q72RC3 | FHA domain-containing protein | ND | ND | [1] |
| LIC11846 | *lic11846* | Q72RA2 | Flagellar motor switch protein FliN | ND | ND | [3] |
| LIC11851 | *impL63* | Q72R97 | Cytoplasmic membrane protein | 666 | ND | [1] |
| LIC11904 | *lic11904* | Q72R45 | Uncharacterized protein | ND | ND | [1] |
| LIC11918 | *lic11918* | Q72R33 | Uncharacterized protein | ND | ND | [1] |
| **LIC11935** | ***lic11935*** | **Q72R17** | **Uncharacterized protein** | **174** | **178** | **[3]** |
| LIC11940 | *lic11940* | Q72R12 | Heavy metal efflux pump | 544 | ND | [1] |
| LIC11941 | *czcC* | Q72R11 | Heavy metal efflux pump | 578 | ND | [1] |
| LIC11946 | *lic11946* | Q72R06 | Thioredoxin domain-containing protein | ND | ND | [1] |
| LIC11959 | *lic11959* | Q72QZ3 | Uncharacterized protein | ND | ND | [1] |
| LIC11975 | *lsa36* | Q72QY3 | Outer membrane protein | ND | ND | [3, 16] |
| LIC11991 | *lic11991* | Q72QW7 | Outer membrane protein | ND | ND | [1] |
| LIC11996 | *erpY-like* | Q72QY9 | Uncharacterized protein | 327 | ND | [1, 17] |
| LIC11997 | *lic11997* | Q72QW1 | Uncharacterized protein | ND | ND | [1] |
| **LIC12002** | ***sdhA*** | **Q72QV6** | **Succinate dehydrogenase flavoprotein subunit** | **97** | **41** | **[3]** |
| LIC12030 | *lic12030* | Q72QS9 | Uncharacterized protein | ND | 179 | [1] |
| LIC12047 | *lic12047* | Q72QR5 | Cytoplasmic membrane protein | ND | ND | [1] |
| LIC12048 | *lic12048* | Q72QR4 | Uncharacterized protein | ND | ND | [1] |
| LIC12067 | *lic12067* | Q72QP6 | Uncharacterized protein | ND | ND | [1] |
| LIC12100 | *lic12100* | Q72QL3 | Uncharacterized protein | 914 | ND | [1] |
| LIC12204 | *lic12204* | Q72QA8 | FAD-dependent oxidoreductase family | ND | ND | [1] |
| LIC12220 | *blaR1* | Q72Q92 | Beta-lactamase regulatory protein 1 | ND | ND | [1] |
| LIC12225 | *lic12225* | Q72Q87 | DUF4105 domain-containing protein | ND | ND | [1] |
| LIC12227 | *lic12227* | Q72Q85 | Uncharacterized protein | ND | ND | [1] |
| LIC12253 | *lic12253* | Q72Q60 | Putative lipoprotein | ND | ND | [3] |
| LIC12254 | *ompL85* | Q72Q59 | Outer membrane protein | 590 | ND | [1, 3, 14] |
| LIC12258 | *lic12258* | Q72Q55 | Omp85 domain-containing protein | ND | ND | [1] |
| LIC12259 | *lic12259* | Q72Q54 | Cytoplasmic membrane protein | ND | ND | [3] |
| LIC12293 | *purK* | Q72Q21 | N5-carboxyaminoimidazole ribonucleotide synthase | ND | ND | [3] |
| LIC12295 | *lic12295* | Q72Q19 | ABC transporter ATP-binding protein | ND | ND | [3] |
| LIC12337 | *lic12337* | Q72PY0 | Uncharacterized protein | ND | ND | [1, 3] |
| LIC12340 | *lic12340* | Q72PX7 | Ricin B-type lectin domain-containing protein | ND | ND | [1] |
| LIC12374 | *btuB* | Q72PU3 | Outer membrane protein, TonB dependent | ND | ND | [1, 3] |
| LIC12499 | *lic12499* | Q72PH2 | Uncharacterized protein | ND | ND | [1, 3] |
| LIC12500 | *mcpB* | Q72PH1 | Chemoreceptor | 542 | ND | [1] |
| LIC12509 | *lic12509* | Q72PG2 | Uncharacterized protein | ND | ND | [3] |
| LIC12518 | *lic12518* | Q72PF3 | Uncharacterized protein | ND | ND | [3] |
| LIC12519 | *lic12519* | Q72PF2 | Uncharacterized protein | ND | ND | [3] |
| LIC12524 | *salD* | Q72PE7 | Fatty acid transport protein | ND | ND | [1] |
| LIC12525 | *lic12525* | Q72PE6 | Putative lipoprotein | ND | ND | [3] |
| LIC12558 | *lic12558* | Q72PB4 | Uncharacterized protein | ND | ND | [3] |
| **LIC12575** | ***lic12575*** | **Q72PA0** | **Cytoplasmic membrane protein** | **197** | **102** | **[1]** |
| **LIC12615** | ***lic12615*** | **Q72P61** | **Phage-related protein** | **251** | **27** | **[1]** |
| LIC12632 | *sph1* | Q72P44 | Sphingomyelinase C 1 | ND | ND | [1] |
| LIC12641 | *lic12641* | Q72P35 | Uncharacterized protein | ND | ND | [1] |
| LIC12649 | *lic12649* | Q72P27 | Uncharacterized protein | ND | ND | [1] |
| LIC12690 | *lic12690* | Q72NY8 | Putative lipoprotein | ND | ND | [1] |
| **LIC12693** | ***lic12693*** | **Q72NY5** | **Uncharacterized protein** | **221** | **182** | **[1]** |
| LIC12709 | *lic12709* | Q72NW9 | Bifunctional NAD(P)H-hydrate repair enzyme | ND | ND | [1] |
| LIC12730 | *lic12730* | Q72NU9 | TPR-REGION domain-containing protein | 832 | ND | [1, 5] |
| LIC12760 | *lic12760* | Q72NR9 | Microbial collagenase | ND | ND | [1] |
| LIC12765 | *tpx* | Q72NR4 | Thiol peroxidase (Tpx) | 479 | ND | [3] |
| LIC12791 | *lic12791* | Q72NP1 | Uncharacterized protein | ND | ND | [1] |
| **LIC12921** | ***mcpA*** | **Q72NB4** | **Methyl-accepting chemotaxis protein** | **21** | **36** | **[1]** |
| LIC12930 | *lic12930* | Q72NA6 | Uncharacterized protein | ND | ND | [3] |
| LIC12936 | *lic12936* | Q72NA0 | Uncharacterized protein | 193 | ND | [1] |
| LIC12952 | *lic12952* | Q72N85 | S-layer-like protein | ND | ND | [1] |
| LIC12985 | *lic12985* | Q72N53 | Uncharacterized protein | ND | ND | [3] |
| LIC12986 | *lic12986* | Q72N52 | Uncharacterized protein | ND | ND | [3] |
| LIC12990 | *lic12990* | Q72N48 | Uncharacterized protein | ND | ND | [1] |
| LIC13000 | *lic13000* | Q72N38 | PpiC domain-containing protein | ND | ND | [1] |
| LIC13002 | *lic13002* | Q72N36 | Uncharacterized protein | ND | ND | [1, 3] |
| LIC13050 | *ompL47* | Q72MY9 | Uncharacterized protein | 800 | ND | [3, 10] |
| LIC13055 | *lic13055* | Q72MY4 | Uncharacterized protein | ND | ND | [1] |
| LIC13060 | *lipL36* | Q72MX9 | LipL36 | 107 | ND | [1] |
| LIC13066 | *lic13066* | Q72MX3 | Putative lipoprotein | ND | 143 | [1, 3] |
| LIC13070 | *lic13070* | Q72MW9 | Uncharacterized protein | ND | ND | [3] |
| LIC13076 | *lic13076* | Q72MW3 | Putative lipoprotein | ND | ND | [1, 3] |
| LIC13078 | *lic13078* | Q72MW1 | Uncharacterized protein | ND | ND | [3] |
| LIC13084 | *lic13084* | Q72MV5 | SPOR domain-containing protein | ND | ND | [1] |
| LIC13086 | *lic13086* | Q72MV3 | Putative lipoprotein | ND | ND | [3] |
| **LIC13089** | ***lic13089*** | **Q72MV0** | **Uncharacterized protein** | **405** | **184** | **[1, 3]** |
| LIC13101 | *lic13101* | Q72MU1 | Outer membrane protein with alpha integrin like repeat domains | ND | ND | [3] |
| LIC13198 | *lic13198* | Q72MI9 | Hemolysin/sphingomyelinase-like | ND | ND | [1] |
| LIC13202 | *choD* | Q72MI5 | Cholesterol oxidase | ND | ND | [1] |
| LIC13215 | *lic13215* | Q72MH2 | Putative lipoprotein | ND | ND | [1] |
| LIC13229 | *lic13229* | Q72MF8 | Alginate_exp domain-containing protein | ND | ND | [1] |
| LIC13231 | *lic13231* | Q72MF6 | FGE-sulfatase domain-containing protein | ND | ND | [1] |
| LIC13238 | *lic13238* | Q72MF0 | Uncharacterized protein | ND | ND | [3] |
| LIC13247 | *lic13247* | Q72ME1 | Uncharacterized protein | ND | ND | [1] |
| LIC13255 | *lic13255* | Q72MD3 | Putative lipoprotein | ND | ND | [3] |
| LIC13305 | *lic13305* | Q72M85 | Putative lipoprotein | ND | ND | [1] |
| LIC13306 | *lic13306* | Q72M84 | Uncharacterized protein | ND | ND | [3] |
| LIC13321 | *lic13321* | Q72M69 | Thermolysin homolog | ND | ND | [1] |
| LIC13322 | *nprT* | Q72M68 | Thermolysin | ND | ND | [1] |
| LIC13334 | *lic13334* | Q72M57 | GMC oxidoreductase | ND | ND | [3] |
| LIC13353 | *lao* | Q72M38 | L-amino acid oxidase | 830 | ND | [1] |
| LIC13354 | *lic13354* | Q72M37 | Uncharacterized protein | 477 | ND | [3] |
| LIC13355 | *lic13355* | Q72M36 | Putative lipoprotein | ND | ND | [3] |
| LIC13381 | *lic13381* | Q72M12 | CrtC domain-containing protein | ND | ND | [1] |
| LIC13386 | *lic13386* | Q72M07 | Uncharacterized protein | ND | ND | [1] |
| LIC13394 | *dmcA* | Q72LZ9 | Methyl-accepting chemotaxis transmembrane protein | ND | ND | [1] |
| LIC13411 | *lic13411* | Q72LY2 | Putative lipoprotein | ND | ND | [1] |
| **LIC13417** | ***lic13417*** | **Q72LX6** | **Alginate_exp domain-containing protein** | **304** | **134** | **[1]** |
| **LIC13418** | ***lic13418*** | **Q72LX5** | **Alginate_exp domain-containing protein** | **487** | **200** | **[3]** |
| **LIC13434** | ***lic13434*** | **Q72LV9** | **Peptidase_M43 domain-containing protein** | **682** | **71** | **[1, 3]** |
| LIC13435 | *lic13435* | Q72LV8 | Uncharacterized protein | ND | ND | [1] |
| LIC13436 | *lic13436* | Q72LV7 | Uncharacterized protein | ND | ND | [3] |
| LIC13447 | *lic13447* | Q72LU6 | Putative lipoprotein | ND | ND | [1] |
| LIC13477 | *lic13477* | Q72LS4 | Uncharacterized protein | 989 | ND | [1, 3] |
| LIC13478 | *lic13478* | Q72LS3 | Cystine-binding periplasmic protein | ND | ND | [1] |
| LIC13479 | *lic13479* | Q72LS2 | Peptidoglycan-associated cytoplasmic membrane protein | ND | ND | [1] |
| **LIC20001** | ***lic20001*** | **Q75G12** | **F5/8 type C domain-containing protein** | **428** | **67** | **[3]** |
| LIC20019 | *lic20019* | Q75FZ6 | Uncharacterized protein | ND | ND | [1] |
| LIC20042 | *batC* | Q75FY9 | BatC | ND | ND | [1, 3] |
| LIC20043 | *batD* | Q75FY8 | BatD | 981 | ND | [1] |
| LIC20056 | *lic20056* | Q75FX5 | Uncharacterized protein | ND | ND | [3] |
| LIC20077 | *lic20077* | Q75FV4 | Polysaccharide deacetylase | ND | ND | [3] |
| LIC20087 | *lic20087* | Q75FU4 | Outer membrane protein | ND | ND | [1, 3] |
| LIC20103 | *lic20103* | Q75FS8 | Uncharacterized protein | ND | ND | [1] |
| LIC20144 | *lic20144* | Q75FN8 | HtrA1-like protein | 154 | ND | [1] |
| LIC20151 | *hbpA* | Q75FN1 | TonB-dependent outer membrane receptor | ND | ND | [1, 3, 18] |
| LIC20152 | *lic20152* | Q75FN0 | Uncharacterized protein | ND | ND | [3] |
| LIC20153 | *lic20153* | Q75FM9 | Uncharacterized protein | ND | ND | [3] |
| LIC20157 | *lic20157* | Q75FM5 | Uncharacterized protein | ND | ND | [1] |
| **LIC20172** | ***lruC, lmb216*** | **Q75FL0** | **Lipoprotein** | **205** | **70** | [1, 19, 20] |
| LIC20185 | *lic20185* | Q75FJ7 | FecR domain-containing protein | 118 | ND | [1, 3] |
| LIC20190 | *lic20190* | Q75FJ2 | Uncharacterized protein | ND | ND | [1] |
| **LIC20197** | ***lic20197*** | **Q75FI7** | **Cysteine protease** | **563** | **139** | **[1, 3]** |
| **LIC20205** | ***lic20205*** | **Q75FI4** | **Rubrerythrin domain-containing protein** | **234** | **74** | **[3]** |
| LIC20212 | *lic20212* | Q75FH7 | Uncharacterized protein | 705 | ND | [3] |
| LIC20214 | *lic20214* | Q75FH5 | Plug domain-containing protein | 299 | ND | [1, 3] |
| LIC20215 | *lic20215* | Q75FH4 | Uncharacterized protein | ND | ND | [3] |
| LIC20231 | *lic20231* | Q75FF9 | Uncharacterized protein | ND | ND | [1] |
| LIC20271 | *lic20271* | Q75FC3 | Uncharacterized protein | ND | ND | [1] |

^a^ The ranking was calculated throughout all identified proteins of surface biotinylation, ND means not detected.

^b^ The ranking was calculated throughout all identified proteins of surface proK shaving, ND means not detected.

Blue fonts imply known SE-OMPs.

Bold fonts imply overlapping proteins.

**Reference**

1. Grassmann AA, Kremer FS, Dos Santos JC, Souza JD, Pinto LDS, McBride AJA. Discovery of Novel Leptospirosis Vaccine Candidates Using Reverse and Structural Vaccinology. Front Immunol. 2017;8:463. Epub 2017/05/13. doi: 10.3389/fimmu.2017.00463. PubMed PMID: 28496441; PubMed Central PMCID: PMCPMC5406399.

2. Cullen PA, Haake DA, Bulach DM, Zuerner RL, Adler B. LipL21 is a novel surface-exposed lipoprotein of pathogenic *Leptospira* species. Infect Immun. 2003;71(5):2414-21. Epub 2003/04/22. doi: 10.1128/iai.71.5.2414-2421.2003. PubMed PMID: 12704111; PubMed Central PMCID: PMCPMC153295.

3. Zeng L, Wang D, Hu N, Zhu Q, Chen K, Dong K, et al. A Novel Pan-Genome Reverse Vaccinology Approach Employing a Negative-Selection Strategy for Screening Surface-Exposed Antigens against leptospirosis. Front Microbiol. 2017;8:396. Epub 2017/03/30. doi: 10.3389/fmicb.2017.00396. PubMed PMID: 28352257; PubMed Central PMCID: PMCPMC5348505.

4. Vieira ML, de Morais ZM, Goncales AP, Romero EC, Vasconcellos SA, Nascimento AL. Lsa63, a newly identified surface protein of *Leptospira interrogans* binds laminin and collagen IV. J Infect. 2010;60(1):52-64. Epub 2009/11/03. doi: 10.1016/j.jinf.2009.10.047. PubMed PMID: 19879894.

5. Vieira ML, Atzingen MV, Oliveira TR, Oliveira R, Andrade DM, Vasconcellos SA, et al. In vitro identification of novel plasminogen-binding receptors of the pathogen *Leptospira interrogans*. PLoS One. 2010;5(6):e11259. Epub 2010/06/29. doi: 10.1371/journal.pone.0011259. PubMed PMID: 20582320; PubMed Central PMCID: PMCPMC2889836.

6. Atzingen MV, Barbosa AS, De Brito T, Vasconcellos SA, de Morais ZM, Lima DM, et al. Lsa21, a novel leptospiral protein binding adhesive matrix molecules and present during human infection. BMC Microbiol. 2008;8:70. Epub 2008/05/01. doi: 10.1186/1471-2180-8-70. PubMed PMID: 18445272; PubMed Central PMCID: PMCPMC2386478.

7. Matsunaga J, Barocchi MA, Croda J, Young TA, Sanchez Y, Siqueira I, et al. Pathogenic *Leptospira* species express surface-exposed proteins belonging to the bacterial immunoglobulin superfamily. Mol Microbiol. 2003;49(4):929-45. Epub 2003/08/02. doi: 10.1046/j.1365-2958.2003.03619.x. PubMed PMID: 12890019; PubMed Central PMCID: PMCPMC1237129.

8. Pinne M, Matsunaga J, Haake DA. Leptospiral outer membrane protein microarray, a novel approach to identification of host ligand-binding proteins. J Bacteriol. 2012;194(22):6074-87. Epub 2012/09/11. doi: 10.1128/JB.01119-12. PubMed PMID: 22961849; PubMed Central PMCID: PMCPMC3486348.

9. Karlsson R, Thorell K, Hosseini S, Kenny D, Sihlbom C, Sjoling A, et al. Comparative Analysis of Two *Helicobacter pylori* Strains using Genomics and Mass Spectrometry-Based Proteomics. Front Microbiol. 2016;7:1757. Epub 2016/11/29. doi: 10.3389/fmicb.2016.01757. PubMed PMID: 27891114; PubMed Central PMCID: PMCPMC5104757.

10. Pinne M, Haake DA. A comprehensive approach to identification of surface-exposed, outer membrane-spanning proteins of *Leptospira interrogans*. PLoS One. 2009;4(6):e6071. Epub 2009/06/30. doi: 10.1371/journal.pone.0006071. PubMed PMID: 19562037; PubMed Central PMCID: PMCPMC2698987.

11. Mendes RS, Von Atzingen M, de Morais ZM, Goncales AP, Serrano SM, Asega AF, et al. The novel leptospiral surface adhesin Lsa20 binds laminin and human plasminogen and is probably expressed during infection. Infect Immun. 2011;79(11):4657-67. Epub 2011/08/17. doi: 10.1128/IAI.05583-11. PubMed PMID: 21844229; PubMed Central PMCID: PMCPMC3257903.

12. Souza NM, Vieira ML, Alves IJ, de Morais ZM, Vasconcellos SA, Nascimento AL. Lsa30, a novel adhesin of *Leptospira interrogans* binds human plasminogen and the complement regulator C4bp. Microb Pathog. 2012;53(3-4):125-34. Epub 2012/06/27. doi: 10.1016/j.micpath.2012.06.001. PubMed PMID: 22732096.

13. Figueredo JM, Siqueira GH, de Souza GO, Heinemann MB, Vasconcellos SA, Chapola EGB, et al. Characterization of two new putative adhesins of *Leptospira interrogans*. Microbiology (Reading). 2017;163(1):37-51. Epub 2017/02/16. doi: 10.1099/mic.0.000411. PubMed PMID: 28198346.

14. Haake DA, Matsunaga J. Leptospira: a spirochaete with a hybrid outer membrane. Mol Microbiol. 2010;77(4):805-14. Epub 2010/07/06. doi: 10.1111/j.1365-2958.2010.07262.x. PubMed PMID: 20598085; PubMed Central PMCID: PMCPMC2976823.

15. Kochi LT, Fernandes LGV, Souza GO, Vasconcellos SA, Heinemann MB, Romero EC, et al. The interaction of two novel putative proteins of *Leptospira interrogans* with E-cadherin, plasminogen and complement components with potential role in bacterial infection. Virulence. 2019;10(1):734-53. Epub 2019/08/20. doi: 10.1080/21505594.2019.1650613. PubMed PMID: 31422744; PubMed Central PMCID: PMCPMC6735628.

16. Siqueira GH, Atzingen MV, Alves IJ, de Morais ZM, Vasconcellos SA, Nascimento AL. Characterization of three novel adhesins of Leptospira interrogans. Am J Trop Med Hyg. 2013;89(6):1103-16. Epub 2013/08/21. doi: 10.4269/ajtmh.13-0205. PubMed PMID: 23958908; PubMed Central PMCID: PMCPMC3854887.

17. Ghosh KK, Prakash A, Dhara A, Hussain MS, Shrivastav P, Kumar P, et al. Role of Supramolecule ErpY-Like Lipoprotein of Leptospira in Thrombin-Catalyzed Fibrin Clot Inhibition and Binding to Complement Factors H and I, and Its Diagnostic Potential. Infect Immun. 2019;87(12). Epub 2019/09/25. doi: 10.1128/IAI.00536-19. PubMed PMID: 31548314; PubMed Central PMCID: PMCPMC6867842.

18. Asuthkar S, Velineni S, Stadlmann J, Altmann F, Sritharan M. Expression and characterization of an iron-regulated hemin-binding protein, HbpA, from *Leptospira interrogans* serovar Lai. Infect Immun. 2007;75(9):4582-91. Epub 2007/06/20. doi: 10.1128/IAI.00324-07. PubMed PMID: 17576761; PubMed Central PMCID: PMCPMC1951163.

19. Verma A, Matsunaga J, Artiushin S, Pinne M, Houwers DJ, Haake DA, et al. Antibodies to a novel leptospiral protein, LruC, in the eye fluids and sera of horses with *Leptospira*-associated uveitis. Clin Vaccine Immunol. 2012;19(3):452-6. Epub 2012/01/13. doi: 10.1128/CVI.05524-11. PubMed PMID: 22237897; PubMed Central PMCID: PMCPMC3294619.

20. Toma C, Murray GL, Nohara T, Mizuyama M, Koizumi N, Adler B, et al. Leptospiral outer membrane protein LMB216 is involved in enhancement of phagocytic uptake by macrophages. Cell Microbiol. 2014;16(9):1366-77. Epub 2014/03/25. doi: 10.1111/cmi.12296. PubMed PMID: 24655538.
