# Supplementary material for: Surface proteomics and label-free quantification of Leptospira interrogans serovar Pomona
Source: PLoS Negl Trop Dis. 2021 Nov 29;15(11):e0009983. doi: 10.1371/journal.pntd.0009983 (PMC8659334; doi:10.1371/journal.pntd.0009983)
Supplement: S3 Table — (DOCX) [file pntd.0009983.s005.docx]

**S3 Table** The most 50 abundant proteins obtained by surface biotinylation and surface shaving.

| Gene ID | Gene name | Protein ID | Protein function | Abundance ranking | |
| --- | --- | --- | --- | --- | --- |
|  |  |  |  | Surface biotinylation^a^ | Surface shaving^b^ |
| LIC10002 | *dnaN* | Q72WD5 | Beta sliding clamp |  | 20 |
| **LIC10011** | ***lipL21*** | **Q72WC6** | **LipL21** | **33** | **13** |
| LIC10175 | *lic10175* | Q72VX0 | Uncharacterized protein |  | 6 |
| LIC10176 | *lic10176* | Q72VW9 | Uncharacterized protein | 24 |  |
| LIC10191 | *loa22* | Q72VV5 | Loa22 | 7 |  |
| LIC10208 | *cyoA* | Q72VT8 | Cytochrome c oxidase subunit 2 | 44 |  |
| LIC10215 | *lic10215* | Q72VT1 | TRAM domain-containing protein | 34 |  |
| LIC10253 | *lic10253* | Q72VP3 | Alcohol dehydrogenase |  | 44 |
| LIC10272 | *fusA* | Q72VM5 | Elongation factor G (EF-G) |  | 30 |
| LIC10314 | *lic10314* | Q72VI3 | Uncharacterized protein |  | 48 |
| LIC10360 | *etfA* | Q72VD8 | Electron transfer flavoprotein alpha-subunit | 27 |  |
| LIC10403 | *ribH* | P61724 | 6,7-dimethyl-8-ribityllumazine synthase (DMRL synthase) | 6 |  |
| **LIC10411** | ***lic10411*** | **Q72V90** | **Uncharacterized protein** | **41** | **47** |
| LIC10443 | *mccB* | Q72V59 | 3-methylcrotonoyl-CoA carboxylase beta subunit |  | 38 |
| LIC10452 | *lic10452* | Q72V51 | Glycosyltransferase | 48 |  |
| LIC10483 | *lic10483* | Q72V20 | Uncharacterized protein | 17 |  |
| LIC10524 | *dnaK* | P61442 | Chaperone protein DnaK |  | 17 |
| LIC10606 | *dps* | Q72UQ1 | DNA-binding stress protein | 38 |  |
| LIC10753 | *rpoB* | Q72UA8 | DNA-directed RNA polymerase subunit beta |  | 15 |
| **LIC10754** | ***rpoC*** | **Q72UA7** | **DNA-directed RNA polymerase subunit beta** | **43** | **10** |
| LIC10787 | *flaA-2* | Q72U75 | Flagellar filament sheath protein | 39 |  |
| LIC10788 | *flaA-1* | Q72U74 | Flagellar filament sheath protein | 25 |  |
| LIC10789 | *greA* | Q72U73 | Transcription elongation factor GreA |  | 34 |
| LIC10842 | *dapA* | Q72U22 | 4-hydroxy-tetrahydrodipicolinate synthase (HTPA synthase) |  | 23 |
| **LIC10874** | ***lic10874*** | **Q72TZ0** | **Molybdopterin oxidoreductase** | **18** | **22** |
| LIC10877 | *lic10877* | Q72TY7 | Cytochrome c domain-containing protein | 49 |  |
| LIC10927 | *lic10927* | Q72TT9 | Putative lipoprotein |  | 21 |
| **LIC10973** | ***ompL1*** | **Q72TP4** | **Outer membrane protein** | **22** | **37** |
| **LIC11003** | ***lipL71*** | **Q72TL5** | **LipL71** | **32** | **7** |
| LIC11013 | *oppA* | Q72TK6 | OppA | 46 |  |
| LIC11182 | *lic11182* | Q72T39 | Uncharacterized protein | 50 |  |
| **LIC11194** | ***lic11194*** | **Q72T27** | **Putative citrate lyase** | **16** | **28** |
| LIC11219 | *ahpC* | Q72T03 | Peroxiredoxin | 26 |  |
| **LIC11241** | ***atpA*** | **Q72SY1** | **ATP synthase subunit alpha (ATP synthase F1 sector subunit alpha)** | **42** | **31** |
| **LIC11243** | ***atpD*** | **Q72SX9** | **ATP synthase subunit beta (ATP synthase F1 sector subunit beta)** | **19** | **25** |
| **LIC11335** | ***groEL*** | **P61438** | **60 kDa chaperonin (GroEL)** | **1** | **2** |
| **LIC11352** | ***lipL32*** | **Q72SM7** | **LipL32** | **2** | **5** |
| LIC11354 | *metK* | Q72SM5 | S-adenosylmethionine synthase (AdoMet synthase) |  | 43 |
| LIC11359 | *maoC* | Q72SM0 | MaoC |  | 9 |
| LIC11456 | *lipL31* | Q72SC8 | LipL31 | 13 |  |
| **LIC11517** | ***accA2*** | **Q72S69** | **Acetyl-CoA carboxylase alpha subunit** | **31** | **8** |
| LIC11531 | *lic11531* | Q72S55 | Flagellin | 37 |  |
| LIC11602 | *lic11602* | Q72RY6 | GSDH domain-containing protein |  | 16 |
| LIC11617 | *lic11617* | Q72RX1 | Transcriptional regulator (ArsR family) | 30 |  |
| LIC11643 | *lic11643* | Q72RU5 | LipL45 | 20 |  |
| LIC11652 | *tal* | Q72RT8 | Probable transaldolase | 11 |  |
| **LIC11687** | ***lic11687*** | **Q72RQ7** | **Endonuclease** | **10** | **45** |
| LIC11701 | *rpoD* | P61540 | RNA polymerase sigma factor RpoD (Sigma-70) |  | 49 |
| LIC11768 | *leuB* | Q72RH7 | 3-isopropylmalate dehydrogenase |  | 40 |
| **LIC11781** | ***mdh*** | **P61975** | **Malate dehydrogenase** | **47** | **12** |
| LIC11793 | *sppA* | Q72RF2 | Signal peptide peptidase | 35 |  |
| LIC11848 | *lic11848* | Q72RA0 | Uncharacterized protein | 36 |  |
| **LIC11885** | ***lipl46*** | **Q72R63** | **LipL46** | **15** | **19** |
| **LIC11890** | ***lic11890*** | **Q72R58** | **Flagellin** | **12** | **26** |
| LIC12002 | *sdhA* | Q72QV6 | Succinate dehydrogenase flavoprotein subunit |  | 41 |
| LIC12017 | *clpB* | Q72QU2 | Chaperone protein (ClpB) |  | 29 |
| LIC12032 | *katE* | Q72QS7 | Catalase |  | 24 |
| **LIC12082** | ***cysK*** | **Q72QN1** | **Cysteine synthase** | **23** | **3** |
| LIC12090 | *gapA* | Q72QM3 | Glyceraldehyde-3-phosphate dehydrogenase |  | 33 |
| LIC12233 | *lic12233* | Q72Q79 | Fructose-bisphosphate aldolase | 9 |  |
| LIC12324 | *lic12324* | Q72PZ1 | Non-specific serine/threonine protein kinase |  | 42 |
| LIC12407 | *glnA* | Q72PR0 | Putative glutamine synthetase protein | 4 |  |
| LIC12476 | *sucB* | Q72PJ5 | Dihydrolipoyllysine-residue succinyltransferase component of 2-oxoglutarate dehydrogenase complex | 28 |  |
| LIC12615 | *lic12615* | Q72P61 | Phage-related protein |  | 27 |
| LIC12621 | *lic12621* | Q72P55 | Uncharacterized protein | 45 |  |
| **LIC12631** | ***lic12631*** | **Q72P45** | **Hemolysin** | **40** | **35** |
| LIC12701 | *pnp* | Q72NX7 | Polyribonucleotide nucleotidyltransferase |  | 46 |
| **LIC12846** | ***rpoA*** | **Q72NI8** | **DNA-directed RNA polymerase subunit alpha** | **29** | **32** |
| **LIC12875** | ***tuf*** | **Q72NF9** | **Elongation factor Tu (EF-Tu)** | **14** | **11** |
| **LIC12921** | ***mcpA*** | **Q72NB4** | **Methyl-accepting chemotaxis protein (McpA)** | **21** | **36** |
| **LIC12966** | ***lipL41*** | **Q72N71** | **LipL41** | **3** | **4** |
| **LIC13166** | ***ompl36*** | **Q72MM7** | **OmpL36** | **8** | **18** |
| LIC13314 | *lic13314* | Q72M76 | ABC transp aux domain-containing protein |  | 50 |
| LIC13328 | *lic13328* | Q72M63 | Isocitrate dehydrogenase |  | 1 |
| LIC13367 | *cysI* | Q72M24 | Sulfite reductase |  | 39 |
| LIC13432 | *lic13432* | Q72LW1 | Uncharacterized protein | 5 |  |
| LIC13470 | *lic13470* | Q72LT0 | Ferredoxin NADP reductase |  | 14 |

^a^ The ranking was calculated throughout all identified proteins of surface biotinylation, ND means not detected.

^b^ The ranking was calculated throughout all identified proteins of surface proK shaving, ND means not detected.

Blue fonts imply known SE-OMPs.

Green fonts imply predicted SE-OMPs.

Bold fonts imply overlapping proteins.
