# Supplementary material for: Surface proteomics and label-free quantification of Leptospira interrogans serovar Pomona
Source: PLoS Negl Trop Dis. 2021 Nov 29;15(11):e0009983. doi: 10.1371/journal.pntd.0009983 (PMC8659334; doi:10.1371/journal.pntd.0009983)
Supplement: S4 Table — (DOCX) [file pntd.0009983.s006.docx]

**S4 Table** Bioinformatics tools used to predict βb-OMP and OM lipoproteins as SE-OMPs

βb-OMP is defined as a protein containing a signal peptide, a transmembrane alpha helix lower than 2, and a beta barrel membrane protein.

| *L. interrogans* serovar Copenhageni | *L. interrogans* serovar Pomona | Signal peptide | | | Transmembrane alpha helix <2 | | | Beta barrel membrane protein | | |
| --- | --- | --- | --- | --- | --- | --- | --- | --- | --- | --- |
|  |  | SignalP | Signal-CF | PrediSi | TMHMM | Phobius | CCTOP | PRED-TMMB | TMBETADISC-RBF | HHomp |
| LIC10175 | LIP3483 | 🗴 | 🗴 | 🗴 | 🗸 | 🗸 | 🗸 | 🗴 | 🗴 | 🗴 |
| LIC10176 | LIP3482 | 🗴 | 🗴 | 🗴 | 🗸 | 🗸 | 🗸 | 🗸 | 🗸 | 🗴 |
| LIC10314 | LIP3334 | 🗸 | 🗸 | 🗸 | 🗸 | 🗸 | 🗸 | 🗴 | 🗸 | 🗴 |
| LIC10411 | LIP3228 | 🗸 | 🗸 | 🗸 | 🗸 | 🗸 | 🗸 | 🗸 | 🗸 | 🗴 |
| LIC10483 | LIP3144 | 🗴 | 🗴 | 🗴 | 🗸 | 🗸 | 🗸 | 🗴 | 🗴 | 🗴 |
| LIC11182 | LIP1773 | 🗴 | 🗴 | 🗴 | 🗸 | 🗸 | 🗸 | 🗸 | 🗴 | 🗴 |
| LIC11848 | LIP2297 | 🗸 | 🗸 | 🗸 | 🗸 | 🗸 | 🗸 | 🗴 | 🗴 | 🗴 |
| LIC12621 | LIP0991 | 🗴 | 🗴 | 🗴 | 🗸 | 🗸 | 🗸 | 🗸 | 🗸 | 🗴 |
| LIC13432 | LIP3761 | 🗸 | 🗸 | 🗸 | 🗴 | 🗴 | 🗴 | 🗴 | 🗸 | 🗴 |

OM lipoprotein is defined as a protein containing a lipoprotein signal peptide and predicted to be in OM.

| *L. interrogans* serovar Copenhageni | *L. interrogans* serovar Pomona | Subcellular localization | | | | LipoP |
| --- | --- | --- | --- | --- | --- | --- |
|  |  | PSORTb | CELLO | GNeg-mPLoc | SOSUI-GramN |  |
| LIC10175 | LIP3483 | Cytoplasmic | Cytoplasmic | Cytoplasmic | Extracellular | 🗴 |
| LIC10176 | LIP3482 | Unknown | Cytoplasmic | Inner membrane | Periplasmic | 🗴 |
| LIC10314 | LIP3334 | Unknown | Outer membrane | Cytoplasmic | Cytoplasmic | 🗴 |
| LIC10411 | LIP3228 | Unknown | Cytoplasmic | Extracellular | Periplasmic | 🗸 |
| LIC10483 | LIP3144 | Unknown | Periplasmic | Inner membrane | Cytoplasmic | 🗴 |
| LIC11182 | LIP1773 | Periplasmic | Periplasmic | Inner membrane | Cytoplasmic | 🗴 |
| LIC11848 | LIP2297 | Unknown | Periplasmic | Inner membrane | Extracellular | 🗸 |
| LIC12621 | LIP0991 | Cytoplasmic | Cytoplasmic | Inner membrane | Periplasmic | 🗴 |
| LIC13432 | LIP3761 | Cytoplasmic | Inner membrane | Inner membrane | Cytoplasmic | 🗴 |
